# The potential global gains in health and revenue from increased taxation of tobacco, alcohol and sugar-sweetened beverages: a modelling analysis

**DOI:** 10.1136/bmjgh-2019-002143

**Published:** 2020-03-29

**Authors:** Amit Summan, Nicholas Stacey, Johanna Birckmayer, Evan Blecher, Frank J Chaloupka, Ramanan Laxminarayan

**Affiliations:** 1 Center for Disease Dynamics, Economics & Policy, Washington, District of Columbia, USA; 2 Priority Cost Effective Lessons for Systems Strengthening, School of Public Health, Faculty of Health Sciences, University of the Witwatersrand, Johannesburg, South Africa; 3 Bloomberg Philanthropies, New York, New York, USA; 4 Health Policy Center, University of Illinois at Chicago, Chicago, Illinois, USA; 5 Princeton Environmental Institute, Princeton University, Princeton, NJ, USA

**Keywords:** health economics, health policy, prevention strategies, public health

## Abstract

**Introduction:**

Globally, a growing burden of morbidity and mortality is attributable to lifestyle behaviours, and in particular to the consumption of tobacco, alcohol and sugar-sweetened beverages (SSB). In low-income and middle-income countries, this increased disease burden falls on already encumbered and resource-constrained healthcare systems. Fiscal policies, specifically taxation, can lower consumption of tobacco, alcohol and SSB while raising government revenues.

**Methods:**

We simulated the health and economic effects of taxing cigarettes, alcohol and SSB over 50 years for 30–79 years old populations using separate mathematical models for each commodity that incorporated country-level epidemiological, demographic and consumption data. Based on data availability, national-level health effects of higher tobacco, alcohol and SSB taxes were simulated in 141, 166 and 176 countries, respectively, which represented 92%, 97% and 95% of the global population, respectively. Economic effects for tobacco, alcohol and SSB were estimated for countries representing 91%, 43% and 83% of the global population, respectively. These estimates were extrapolated to the global level by matching countries according to income level.

**Results:**

Over 50 years, taxes that raise the retail price of tobacco, alcoholic beverages and SSB by 20% could result in a global gain of 160.7 million (95% uncertainty interval (UI): 96.3 to 225.2 million), 227.4 million (UI: 161.2 to 293.6 million) and 24.3 million (UI: 15.7 to 35.4 million) additional life years, respectively.

**Conclusion:**

Excise tax increases on tobacco, alcohol and SSB can produce substantial health gains by reducing premature mortality while raising government revenues, which could be used to increase public health funding.

**Key questions**

**What is already known?**

- Fiscal policy tools remain underused in reducing consumption of unhealthy commodities that harm human health.
- The literature has highlighted the economic and health benefits of taxing tobacco, alcohol and sugar-sweetened beverages.
- Modelling for taxes on these commodities is generally conducted for individual countries using varying methodologies, yielding results that may not be comparable; many countries lack analyses altogether.

**What are the new findings?**

- This study provides a global estimate of the impact of raising tobacco, alcohol and sugar-sweetened beverage taxation using a consistent model framework.
- All countries, especially low-income and middle-income countries, can benefit substantially by raising taxes on alcohol and tobacco, and can derive large benefits from taxation of sugar-sweetened beverages.

**What do the new findings imply?**

- Countries should increase tobacco and alcohol taxes and introduce taxes on sugar-sweetened beverages within local tax frameworks to curb consumption of harmful commodities.
- Complementary policies—consumer education, subsidisation of more healthful alternatives, tools to help reduce consumption, restrictions on the use or sale of these commodities—should be deployed to complement taxation measures and can be funded by increased government receipts from taxation.

## Introduction

People living in low-income countries (LIC) and middle-income countries face a growing burden of non-communicable disease attributable to rising consumption of tobacco, alcohol and sugars. To varying degrees, taxes on these commodities have helped reduce their consumption and the associated disease burden while simultaneously raising revenue for governments. However, rates and structures of taxation vary widely by country and are low in many parts of the world where consumption of the harmful commodity is increasing. In this study, we simulated the global effects of increased taxes to curb the burdens of tobacco smoking, alcohol consumption and sugar-sweetened beverage (SSB) consumption on health, expenditure and tax revenue outcomes.

Globally, tobacco smoking ranked as the fourth highest risk factor for years of life lost (YLL) and was responsible for 175 million YLL in 2017.^1^ A recent meta-analysis found that there is no safe level of alcohol consumption^2^; alcohol consumption was responsible for 88 million YLL globally in 2017 and was ranked as the highest risk factor for individuals between the ages of 15 and 49.^1^ Alcohol consumption projections up to 2025 suggest an increase in alcohol consumption in half of all WHO regions.^3^ Excess consumption of SSBs has been linked to obesity and diabetes,^4 5^ whose prevalences are rising around the world, including in LIC and middle-income countries.^6 7^ Growing incomes in these countries are making these commodities more affordable, thus leading to higher levels of consumption.

Fiscal policies, in particular excise taxes, play a large but underappreciated role in improving population health. The Lancet Commission on Investing in Health in 2013 pointed out that ‘fiscal policies are a powerful and underused lever for curbing non-communicable diseases and injuries’.^8^ WHO recognises excise taxes as effective tools to curb harmful alcohol, tobacco and SSB consumption.^9 10^ Fiscal measures offer an appealing complementary opportunity to improve health by modifying risk factors without requiring additional budgetary allocations to ministries of health.^9 11 12^ Revenues raised through taxes could subsidise health expenditures or offset other sources of revenue for national governments.^13–15^ Fiscal policies can encourage healthy behaviours by modifying incentives for treating and preventing illness and making better lifestyle choices, with important implications for public health expenditure and for the large out-of-pocket health expenditures incurred in the private sector.

Taxes on tobacco, alcohol and SSB can also facilitate universal health coverage, which is now part of the United Nations’ (UN) sustainable development goals. In countries as diverse as South Africa, India and Brazil, progress on universal healthcare has run up against the barrier of high rates of smoking and high consumption of alcohol and SSBs, all of which hinder efforts to improve health. Slowing economic growth has reduced government revenues in middle-income countries. Annual growth rates in Brazil, Russia, India, China and South Africa were a population-weighted average of more than two percentage points lower during 2011–2018 than during the previous decade.^16^ The fiscal space for health, already constrained by the low-tax base in many countries, has narrowed further. Moreover, availability of resources does not necessarily lower the disease burden if a country’s health system is weak. In such cases, taxes and subsidies that prevent disease could potentially be more effective than treatment in a poor healthcare system.

In this study, we use a consistent modelling approach to develop global estimates of the effects of fiscal policy tools across three important modifiable risk factors, consumption of: tobacco, alcoholic beverages and SSBs. We synthesise existing evidence on product use, risk factor prevalence, price responsiveness and mortality risks at the country level to simulate the effect of excise tax policies globally on comparable population health outcomes and government revenue estimates.

## Method

### Modelling approach

#### Overview

We simulated the health and economic effects of tobacco, alcohol and SSB taxation using separate mathematical models for each commodity that incorporated country-level epidemiological, demographic and consumption data. Model outputs included years of life gained (YLG), (premature) deaths averted, change in consumer spending and change in tax revenue. Outcomes were aggregated and presented by World Bank country income group classifications: LIC, lower middle-income countries (LMIC), upper middle-income countries (UMIC) and high-income countries (HIC).

**Figure 1:**
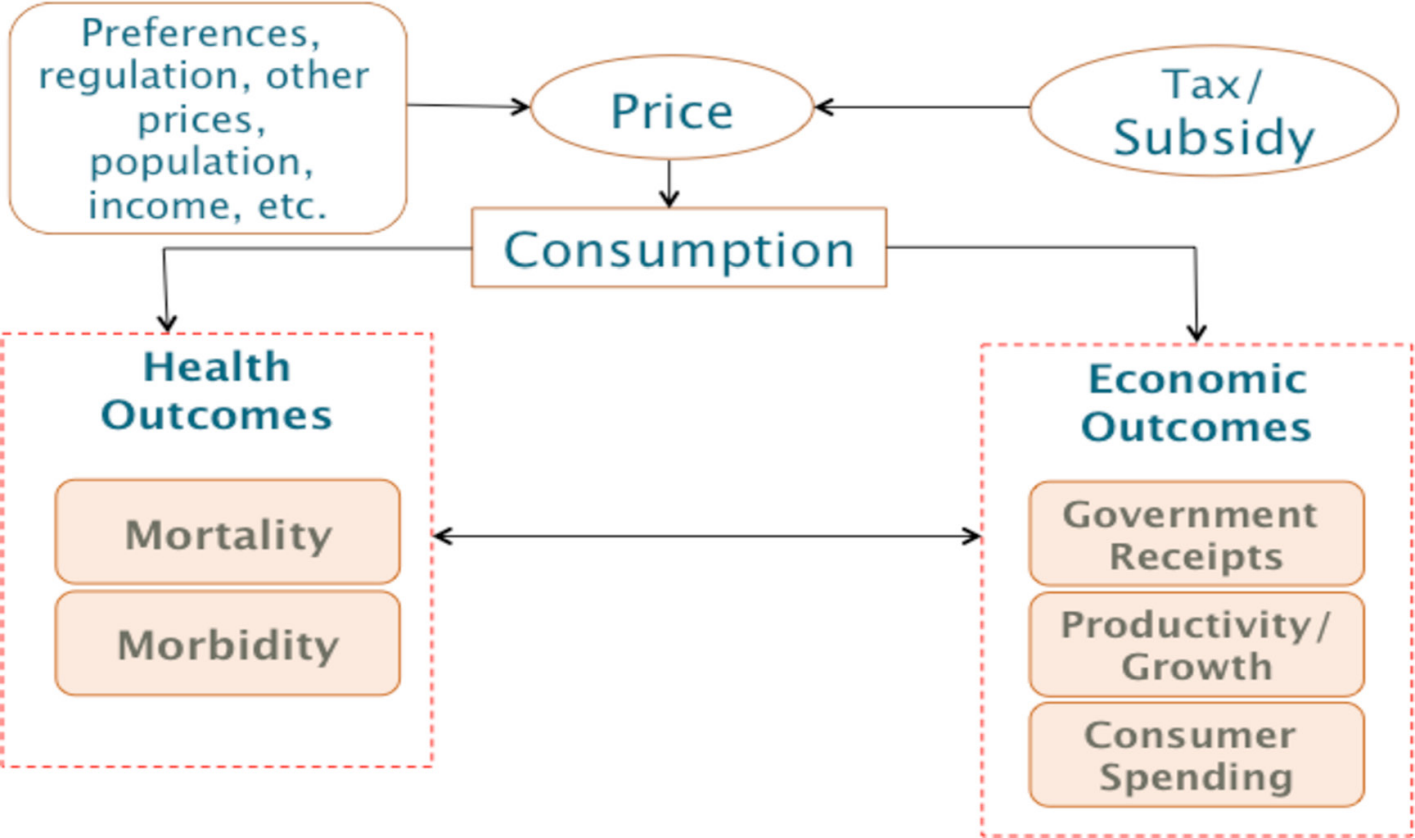
Conceptual framework of modelling. Arrows indicate the direction of causation.

Figure 1 lays out the conceptual structure of the model. The tax is applied to the targeted product, which leads to a price increase and reduces consumption. Prices may change less than projected if the producer absorbs some or all of the costs of the tax. We use a common assumption^14 15 17^ of a 100% pass through of the tax, discussed in the online supplementary appendix. Reduced consumption changes the distribution of risk factors associated with the product within affected populations, ultimately affecting health outcomes. The magnitude of the change in consumption due to the price increase is determined by price elasticities of demand. The taxes also have direct economic consequences for consumer expenditures and for government receipts, as well as indirect outcomes that can include economic growth and labour outcomes.

10.1136/bmjgh-2019-002143.supp1 Supplementary data

We modelled two scenarios, a 20% and a 50% price increase through tax increases. The models focused on health effects for directly exposed individuals but did not consider externality effects of use, such as secondhand smoke or secondary consequences of alcohol and SSB consumption. The time horizon for the model was 50 years, with 2018 as the baseline year. A detailed mathematical description of the model is provided in the online supplementary appendix. The models were implemented in Microsoft Excel 2016 (with visual basic for applications) and R software (R 3.5.3).

#### Health outcomes

In the baseline scenario, future consumption per capita for each commodity for each country was estimated using published consumption projections—from WHO for tobacco^18^ and alcohol^19^ and from Euromonitor^20^ for SSBs. Because projections were not available for all countries, an average trend was calculated for each income group category and applied to countries that were missing a trend. For SSBs, consumption trend projections were not available for any LIC; we used the estimated LMIC consumption trend for LIC in our modelling. These trends were used to estimate a baseline consumption trajectory for 5–15 years. We assumed that consumption growth was flat from year 16 to year 50.

We simulated the health effects of the intervention using a standard, abridged baseline life table (for information on the construction of life tables, see UN^21^ or Gardner and Stewart^22^) and estimated an intervention life table with modified mortality rates. Five-year age intervals were used from ages 15 to 79; the last age category consisted of individuals 80 years and older. Following other modelling literature,^14^ we assumed that cohorts born after 2018 were the same size as the current newborn to 5-year-old cohort. Health effects were estimated for populations above the age of 30 according to the data availability of mortality rates and risks for consumption of the commodities modelled. We focused on the direct association of consumption of the three commodities and all-cause mortality.

#### Economic outcomes

To simulate economic outcomes, we calculated changes in consumer spending and tax revenue for each country over a 50-year period. Consumption levels and patterns (prevalence) were calculated at the beginning of each 5-year period and assumed to be unchanged for this period. All results are in 2018 USD, converted at current exchange rates. Future estimates of expenditures and revenues were discounted using a constant rate of 3%.

#### Sensitivity analysis

We conducted a sensitivity analysis using the Latin hypercube sampling method^23^ on all estimates by drawing independent samples of parameters of elasticity and relative risk, varying them between 20% above and below their mean value using a uniform distribution for each type of product for 1000 iterations. We report the mean value of the resulting distribution as the point estimate, and the 2.5th percentile and 97.5th percentile are provided as a 95% uncertainty interval (UI). Table 1 shows parameter values and table 2 shows input sources. The next sections discuss methodological detail specific to the modelling of each commodity and the resulting global estimates.

**Table 1:** Model parameters

Variable		Data source	Value
*Tobacco*			
Own-price elasticity	Cigarettes: LIC, LMIC, UMIC	Authors’ assumptions based on International Agency for Research on Cancer^44^	–0.5
	Cigarettes: HIC		–0.4
Relative risk of all-cause mortality	Cigarette smoker	Authors’ assumptions based on multiple sources (see table A3 in online supplementary appendix)	2.2
	Former smoker	Authors’ assumption based on Doll *et al*^28^	See figure 4 in Doll *et al*^28^
*Alcohol*			
Own-price elasticity	Alcohol: LIC, LMIC, UMIC	Authors’ assumption based on Nelson^45^	–0.65
Relative risk of all-cause mortality	Daily consumption of grams of pure alcohol	Authors’ estimates based on Grisworld *et al*^46^	See figure A2 in online supplementary appendix
*Sugar-sweetened beverages*			
Own-price elasticity	Sugar-sweetened beverages	Authors’ estimates based on Cabrera Escobar *et al*^5^	–1.2
Relative risk of all-cause mortality	Body mass index	Authors’ estimates based on Aune et al^47^	See figure A3 in online supplementary appendix

HIC, high-income country; LIC, low-income country; LMIC, lower middle-income country; UMIC, upper middle-income country.

**Table 2:** Input sources

Input	Source
Baseline mortality rates	Global Burden of Disease^1^
Population	United Nations World Population Prospects^48^
Income groups	World Bank^36^
*Tobacco*	
Prices	WHO^24^
Tax rates	
Smoking prevalence and trends	WHO^18^
Smoking death rates	Global Burden of Disease^1^
Cigarette consumption	Euromonitor^20^; Ng *et al*^49^
*Alcohol*	
Prices	WHO^50^; Euromonitor^20^; OECD^51^
		Tax rates
Drinking prevalence and trends		WHO Global Health Observatory^50^
Grams of pure alcohol consumption		
Alcohol death rates	Global Burden of Disease^1^
*Sugar-sweetened beverages*	
Prices	Blecher *et al*^52^; Euromonitor^20^
Consumption	Singh *et al*^53^
Overweight and obese prevalence	Global Burden of Disease^54^
Height	NCD Risk Factor Collaboration^55^
Calorie consumption trends	Food and Agriculture Organization^32^

NCD, non-communicable disease; OECD, Organisation for Economic Cooperation and Development.

### Tobacco taxation

We focused on taxation of cigarettes, the most commonly consumed tobacco product, in our tobacco model—the average proportion of daily cigarette smoker prevalence to daily tobacco smoker prevalence across countries is 86%.^24^ The price change induced by a tax increase was assumed to reduce demand for cigarettes at both the extensive margin (number of smokers) and the intensive margin (number of cigarettes smoked by each smoker). Following the tobacco excise modelling literature, we assumed that the overall price elasticity of tobacco was split evenly between changes in the number of current smokers and changes in the intensity of consumption by continuing smokers. We assumed that the elasticity for younger groups, ages 15–25, was twice as large as for other age groups, in line with other studies.^14 15^ In the model, the number of current smokers fell in response to the higher tax because of decreased initiation and increased cessation, raising the number of both former and never smokers. The health effects in our model were attributable to changes in smoking status and not to changes in the intensity of smoking.

Following earlier literature,^13 17 25–27^ we built on a commonly used multistate life table modelling approach, whereby we estimated separate life tables for smokers, never smokers and current former smokers (smokers who quit smoking before the intervention) under a baseline scenario, and intervention life tables for never smokers, current smokers, current former smokers and intervention former smokers (smokers who quit because of the intervention). Successive age–sex cohorts were fed into the country-specific life table structures, and the number of deaths and years of life were calculated in both the baseline and the intervention scenario over a 50-year period.

To account for the benefits of smoking cessation, we used relative risk estimates for former smokers from a study of British doctors.^28^ This study of the long-term effects of smoking on mortality found that smoking cessation by age 30 helped avoid almost all of the excess risk of smoking on average, and that lifelong smokers lost approximately 10 years of healthy life compared with lifelong non-smokers.

### Alcohol taxation

Following previous alcohol modelling,^29^ changes in the price of alcohol from higher taxation were modelled to explore the effect on drinking intensity. We considered three beverage categories: spirits, wine and beer. We did not model substitution because of inconsistent evidence on cross-price elasticities between beverages. Additionally, we simulated the tax increases on each beverage that would lead to a uniform price increase across all three beverage categories.

To estimate the health effects, we used a similar approach to that used for tobacco and constructed separate life tables for drinkers and abstainers. We accounted for the time lag between reduced alcohol consumption and the reduced risk of chronic alcohol-related diseases using published estimates^30^ on the temporal relationship between alcohol consumption and harm for 23 chronic diseases. A detailed description is provided in section 5.3 of the online supplementary appendix.

### SSB taxation

To model SSB taxation, we adopted an energy-balance approach to simulating shifts in body mass index (BMI) distribution associated with changes in beverage intake.^5 13 17^ A previously estimated factor converting average energy imbalance to change in average body weight, 94 kJ/kg, was used to simulate a change in average BMI for each age–sex group.^31^ Data from the Global Burden of Disease study on the prevalence of obesity (BMI >30 kg/m^2^), and obesity and overweight (BMI >25 kg/m^2^) were used for all the countries under consideration to construct baseline log-normal BMI distributions. We then resimulated BMI distributions, accounting for body weight changes arising from underlying country-specific trends in energy intake as well as changes in energy intake that might result from the tax. Individuals with a BMI of less than 24 were assumed to fully offset the decrease in SSB consumption due to the tax with other calories. To account for changes in total calories under the baseline scenario, we incorporated estimates of the change in total calorie consumption from the Food and Agriculture Organization,^32^ which projected total changes in calorie consumption by region to 2050.

To translate changes in age–sex BMI distributions into changes in age–sex mortality rates, we calculated a potential impact fraction, which measures the proportional change in risk due to changing risk factor distribution. This measure is used to scale the prevailing mortality risk in the baseline life table so that an intervention life table can be constructed. Similar to the tobacco and alcohol models, for SSB consumption we applied the overall relationship between all-cause mortality and exposure (BMI-related diseases include stroke, ischaemic heart disease, hypertensive heart disease, diabetes mellitus, osteoarthritis, postmenopausal breast cancer, colon cancer, endometrial cancer and kidney cancer^33^) to model health effects. We assumed the full benefits of reduced SSB consumption accrue starting 5 years after the tax is implemented. This corresponds to the time it takes for an individual to reach a new equilibrium weight after reducing calories because of the intervention.

For the SSB tax modelling, additional consumption of alternative beverages or foods was not explicitly modelled because of a lack of data on cross-price elasticities, but a 50% offset factor was assumed to account for substitution of calories. A 50% offset factor means that half of the reduced calories from decreased SSB consumption are replaced by calories from other beverages. Also because of a lack of data, we did not include the additional expenditure or tax revenue generated from consumption of substituted products in the expenditure and tax estimates. The use of a 50% offset factor is slightly conservative but consistent with the levels of substitution used in other SSB tax modelling,^34 35^ where a roughly 40% offset factor has been used.

### Global effects

To estimate global health effects, we estimated the total health effects per 100 000 individuals by each country income group, using the four World Bank income group classifications.^36^ We then matched countries not covered in our sample by income level to these estimates and imputed the in-sample, weighted health effects to these countries. Parameter and input data were available for countries across all three commodities for estimating health effects: tobacco data were available for countries accounting for 92% of the global population; alcohol data, 97%; and SSBs, 95%. To estimate global economic effects, we first matched countries by exposure level (tertiles of smoking prevalence for tobacco, daily alcohol consumption for alcohol and daily SSB consumption for SSBs), region and income. We then imputed in-sample average missing economic parameter data (price and tax) for countries that were missing these data but had underlying consumption pattern data and simulated the economic effects for these countries. For countries missing both economic and consumption data, we imputed the population-weighted average economic effects calculated at the country’s income level. Tobacco and SSB economic parameter data were available for countries representing 89% and 83% of the global population, respectively. For alcohol, our sample covered 72% and 43% of the global population for consumer expenditure and tax revenue estimates, respectively. For SSBs, we did not have economic parameter data for any LIC which represent 9% of the global population; therefore, for extrapolation to LIC, we used LMIC estimates. (We also estimated economics effects for LIC using estimates from LMIC that represent the bottom 50% of income; we found no significant difference in estimates.) The countries included in the sample for different models are listed in table A5 in the online supplementary appendix. The availability of data for a large number of countries decreases the potential error from extrapolation to the global level.

### Patient and public involvement

This research was done without patient involvement. Patients were not invited to comment on the study design and were not consulted to develop patient relevant outcomes or interpret the results. Patients were not invited to contribute to the writing or editing of this document for readability or accuracy.

## Results

### Tobacco taxation

Table 3 presents the results for tobacco taxation by country income level. For 20% and 50% increases in the price, we estimated YLG of 161 million (95% UI: 96 to 225 million) and 402 million (UI: 241 to 563 million), respectively, over 50 years globally. The health gains from the tax come mainly from LMIC and UMIC; HIC account for the smallest gains across all simulations. We have also presented YLG per 100 000 people across simulations to account for population distribution across income groups. Whereas LIC make up lower total YLG than HIC, the per capita health effects for LIC are higher than HIC—in the 20% price increase simulation, LIC and HIC have YLG of 1836 (UI: 1059 to 2462) and 1226 (UI: 695 to 1657) per 100 000 people, respectively.

**Table 3:** Effects of tobacco taxation over 50 years

Income level	Deaths averted (000s)	Years of life gained (000s)	Deaths averted (per 100 000)	Years of life gained (per 100 000)	Change in expenditure (discounted, US$ 2018, billions)	Change in tax revenue (discounted, US$ 2018, billions)
20% price increase						
Low	722	12 007	110	1836	9	27
	(440 to 1004)	(7224 to 16 790)	(67 to 153)	(1105 to 2568)	(3 to 15)	(23 to 29)
Lower middle	3686	64 881	123	2170	147	277
	(2235 to 5137)	(38 922 to 90 840)	(75 to 172)	(1302 to 3038)	(70 to 205)	(213 to 306)
Upper middle	3535	69 373	138	2711	427	857
	(2135 to 4934)	(41 610 to 97 135)	(83 to 193)	(1626 to 3796)	(189 to 658)	(690 to 1013)
High	739	14 474	63	1226	556	826
	(438 to 1040)	(8556 to 20 391)	(37 to 88)	(725 to 1728)	(374 to 727)	(688 to 949)
Global	8682	160 734	118	2177	1139	1987
	(5249 to 12 115)	(96 312 to 225 157)	(71 to 164)	(1305 to 3050)	(636 to 1606)	(1613 to 2297)
50% price increase						
Low	1805	30 018	276	4591	7	49
	(1101 to 2509)	(18 061 to 41 976)	(168 to 384)	(2762 to 6420)	(−10 to 24)	(36 to 58)
Lower middle	9215	162 202	308	5425	188	497
	(5587 to 12 844)	(97 304 to 227 100)	(187 to 430)	(3254 to 7595)	(−11 to 364)	(325 to 606)
Upper middle	8836	173 432	345	6778	481	1506
	(5339 to 12 334)	(104 025 to 242 839)	(209 to 482)	(4065 to 9490)	(−172 to 1127)	(1009 to 1982)
High	1848	36 184	157	3066	924	1573
	(1096 to 2600)	(21 390 to 50 978)	(93 to 220)	(1812 to 4319)	(414 to 1417)	(1164 to 1953)
Global	21 705	401 836	294	5443	1601	3625
	(13 123 to 30 287)	(240 779 to 562 892)	(178 to 410)	(3261 to 7624)	(220 to 2933)	(2534 to 4599)

Uncertainty interval (95%) in parentheses. Discount rate of 3% is assumed for economic outcomes.

**Figure 2:**
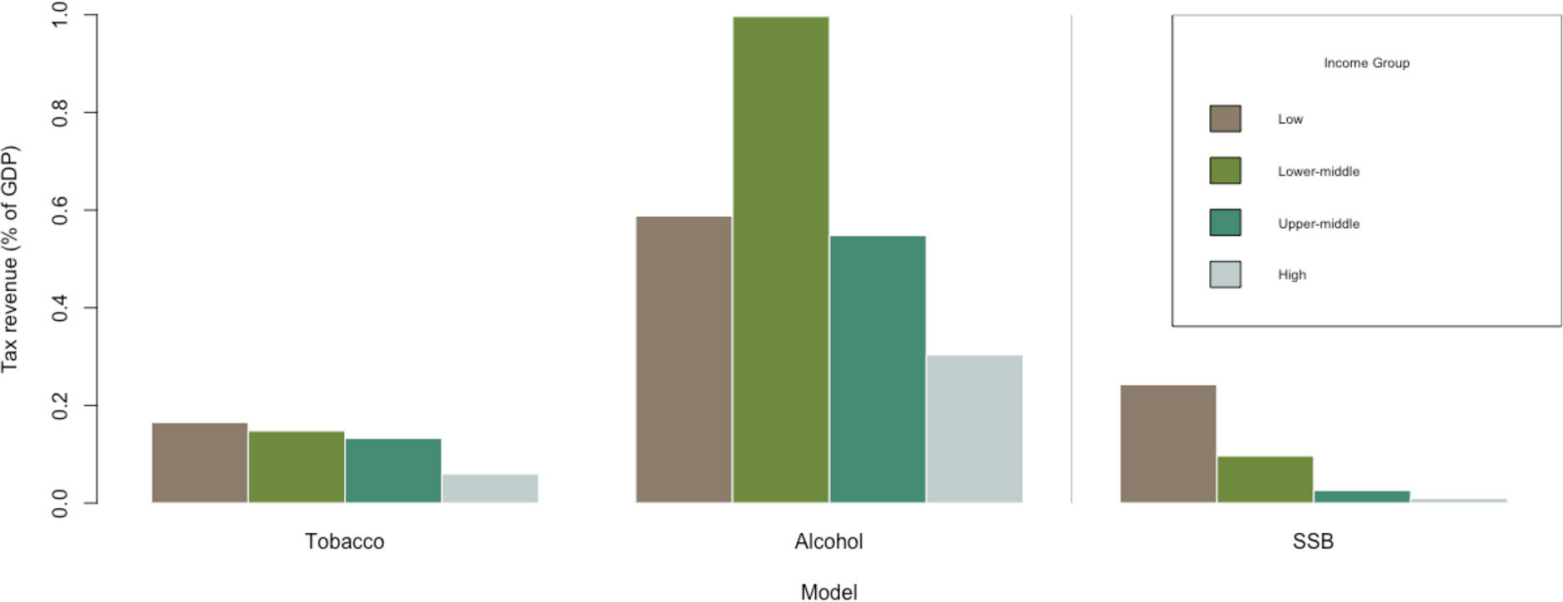
Change in annual tax revenue as percentage of 2018 gross domestic product (GDP). Calculated by dividing total tax revenue by 50 and dividing by 2018 GDP. Source: authors’ estimates. SSB, sugar-sweetened beverages.

Consumer expenditure increases by US$1139 billion (UI: US$636 to US$1606 billion) and US$1601 billion (UI: US$220 to US$2933 billion) over 50 years, and tax revenues increase by US$1987 billion (UI: US$1613 to US$2297 billion) and US$3625 billion (UI: US$2534 to US$4599 billion) in the 20% and 50% price increase simulations, respectively. Figure 2 shows the increase in average annual tax revenue as a percentage of gross domestic product (GDP) in 2018. Although most of the global tax revenue gains come from HIC, the increase in tax revenue as a percentage of GDP is inversely proportional to income level, with LIC increasing their average annual tax revenue by 0.17% as a percentage of GDP in the 50% price increase scenario.

### Alcohol taxation

Table 4 shows results for alcohol taxation by income level. We estimated a health benefit of YLG over 50 years of 227 million (UI: 161 to 294 million) and 547 million (UI: 391 to 703 million) for the 20% and 50% price increases, respectively. LMIC make up 44% of global YLG in both price increase scenarios. LIC have the lowest consumption rate of all the income groups, and thus a low share of global YLG. The population-weighted effects of the tax are highest for HIC, with YLG of 9062 (UI: 6333 to 11 792) per 100 000 people, followed by LMIC, UMIC and LIC in the 50% price increase scenario.

**Table 4:** Effects of alcohol taxation over 50 years

Income level	Deaths averted (000s)	Years of life gained (000s)	Deaths averted (per 100 000)	Years of life gained (per 100 000)	Change in expenditure (discounted, US$ 2018, billions)	Change in tax revenue (discounted, US$ 2018, billions)
20% price increase						
Low	591	11 665	90	1784	37	98
	(435 to 747)	(8355 to 14 975)	(64 to 110)	(1225 to 2196)	(15 to 59)	(87 to 109)
Lower middle	4179	98 999	140	3311	548	1767
	(3095 to 5262)	(71 194 to 126 804)	(99 to 169)	(2283 to 4066)	(223 to 872)	(1654 to 1880)
Upper middle	2460	70 097	96	2739	1013	3291
	(1780 to 3141)	(49 382 to 90 812)	(67 to 118)	(1850 to 3403)	(404 to 1623)	(3079 to 3503)
High	1536	46 661	130	3954	1360	4272
	(1085 to 1987)	(32 295 to 61 026)	(88 to 161)	(2624 to 4958)	(556 to 2163)	(3983 to 4561)
Global	8766	227 421	119	3080	2958	9428
	(6395 to 11 136)	(161 226 to 293 617)	(83 to 145)	(2094 to 3813)	(1198 to 4718)	(8803 to 10 053)
50% price increase						
Low	1431	28 139	210	4126	20	174
	(1059 to 1803)	(20 300 to 35 978)	(155 to 264)	(2977 to 5276)	(−48 to 87)	(133 to 214)
Lower middle	10 101	237 981	324	7631	293	3353
	(7527 to 12 674)	(172 499 to 303 463)	(241 to 406)	(5532 to 9731)	(−710 to 1297)	(2883 to 3823)
Upper middle	5957	169 077	223	6335	520	6229
	(4337 to 7577)	(120 008 to 218 147)	(163 to 284)	(4497 to 8174)	(−1363 to 2404)	(5347 to 7110)
High	3687	111 548	300	9062	716	8022
	(2625 to 4749)	(77 949 to 145 146)	(213 to 386)	(6333 to 11 792)	(−1780 to 3212)	(6825 to 9220)
Global	21 176	546 745	275	7101	1549	17 778
	(15 548 to 26 804)	(390 755 to 702 735)	(202 to 348)	(5075 to 9126)	(−3902 to 6999)	(15 188 to 20 367)

Uniform price increase across all beverages, no substitution assumed. Uncertainty interval (95%) in parentheses. Discount rate of 3% is assumed for economic outcomes.

Consumer expenditure increases by US$2958 billion (UI: US$1198 to US$4718) and US$1549 billion (UI: –US$3902 to US$6999 billion) over 50 years, and tax revenues increase by US$9428 billion (UI: US$8803 to US$10 053 billion) and US$17 778 billion (UI: US$15 188 to US$20 367 billion) for the 20% and 50% price increases, respectively. The increases in both consumer expenditures and tax receipts are largely driven by UMIC and HIC, likely because of these countries’ higher intensity of consumption and higher prices for alcoholic beverages (both daily consumption and prices are highest in HIC). Figure 2 shows that as a percentage of GDP, the annual average tax increases are highest for LMIC, with substantial gains for UMIC and LIC as well.

A challenge with the alcohol expenditure and tax analysis was the lack of price and tax data, particularly in LIC. For 24 LIC, there were parameter data available for two of the three alcoholic beverages; missing tax and price data for the third alcoholic beverage were imputed using average parameter values across LIC for which data were available for the respective beverage type.

Another challenge with alcohol modelling relates to substitution across beverages. Evidence on cross-price elasticities was insufficient, particularly for LMIC, so we used a uniform price increase across beverage types. This limitation is described further in the Discussion section.

### SSB taxation

We simulated tax increases leading to 20% and 50% price increases in SSBs, assuming a 50% offset factor to account for substitution towards other beverages or foods due to a tax. Table 5 shows results for SSB taxation by income level. The 20% and 50% price increases result in YLG of 24 million (UI: 16 to 35 million) and 60 million (UI: 39 to 86 million) over 50 years, respectively. These gains are mostly driven by LMIC and UMIC, which together contribute 45% and 38% to overall YLG in the 20% and 50% price simulations, respectively. These results are consistent with consumption patterns: UMIC have the highest levels of consumption, followed by LMIC and UIC, which have similar levels of consumption. The population-weighted health effects are more equal across income groups and highest for HIC.

**Table 5:** Effects of sugar-sweetened beverage taxation over 50 years

Income level	Deaths averted (000s)	Years of life gained (000s)	Deaths averted (per 100 000)	Years of life gained (per 100 000)	Change in expenditure (discounted, US$ 2018, billions)	Change in tax revenue (discounted, US$ 2018, billions)
20% price increase						
Low	68	1831	10	268	−32	55
	(39 to 104)	(1173 to 2666)	(6 to 15)	(172 to 391)	(−51 to −12)	(51 to 58)
Lower middle	347	8655	11	278	−143	247
	(216 to 511)	(5580 to 12 586)	(7 to 16)	(179 to 404)	(−232 to −54)	(232 to 262)
Upper middle	315	8993	12	337	−130	225
	(201 to 466)	(5799 to 13 019)	(8 to 17)	(217 to 488)	(−211 to −49)	(212 to 239)
High	143	4876	12	396	−114	197
	(87 to 214)	(3132 to 7087)	(7 to 17)	(254 to 576)	(−184 to −43)	(185 to 208)
Global	873	24 355	11	316	−419	724
	(544 to 1295)	(15 684 to 35 358)	(7 to 17)	(204 to 459)	(−679 to −158)	(680 to 767)
50% price increase						
Low	188	4519	28	663	−144	72
	(116 to 281)	(2920 to 6549)	(17 to 41)	(428 to 960)	(−205 to −82)	(51 to 92)
Lower middle	872	21 354	28	685	−650	325
	(558 to 1282)	(13 823 to 30 929)	(18 to 41)	(443 to 992)	(−927 to −372)	(232 to 417)
Upper middle	796	21 863	30	819	−593	297
	(510 to 1161)	(14 224 to 31 427)	(19 to 44)	(533 to 1178)	(−847 to −339)	(212 to 381)
High	377	12 026	31	977	−517	259
	(235 to 556)	(7766 to 17 382)	(19 to 45)	(631 to 1412)	(−739 to −296)	(185 to 333)
Global	2234	59 762	29	776	−1903	952
	(1419 to 3281)	(38 732 to 86 287)	(18 to 43)	(503 to 1121)	(-2717 to −1090)	(681 to 1224)

A 50% offset factor is assumed. Uncertainty interval (95%) in parentheses. Discount rate of 3% is assumed for economic outcomes.

The 20% and 50% price increases result in tax revenue gains of US$724 billion (UI: US$680 to US$767 billion) and US$952 billion (UI: US$681 to US$1224 billion) over 50 years, respectively. (We found that the lower bound for the 95% CI for the 20% and 50% price increase simulations is similar as the reduction in consumption in the 50% price increase simulation is compensated by the new tax revenue, given baseline parameters, to mathematically be close to the lower bound for the 20% price increase simulation on average.) The corresponding consumer expenditure changes are –US$419 billion (UI: –US$679 to –US$158 billion) and –US$1903 billion (UI: –US$2717 to –US$1090 billion). Demand for SSBs, unlike the other commodities, is elastic, causing a decrease in consumption expenditure for consumers. (We did not make an assumption about the cost of the good substituted towards when estimating consumer expenditure and therefore do not include expenditure on substituted goods in our estimates.) Tax revenue contributions and decreases in consumer expenditure shares are greater than the share of population for HIC, reflecting these countries’ high level of consumption and high average SSB prices. However, figure 2 shows that average annual tax revenue gains as a percentage of GDP are inversely related to income level.

### Trends in health outcomes

**Figure 3:**
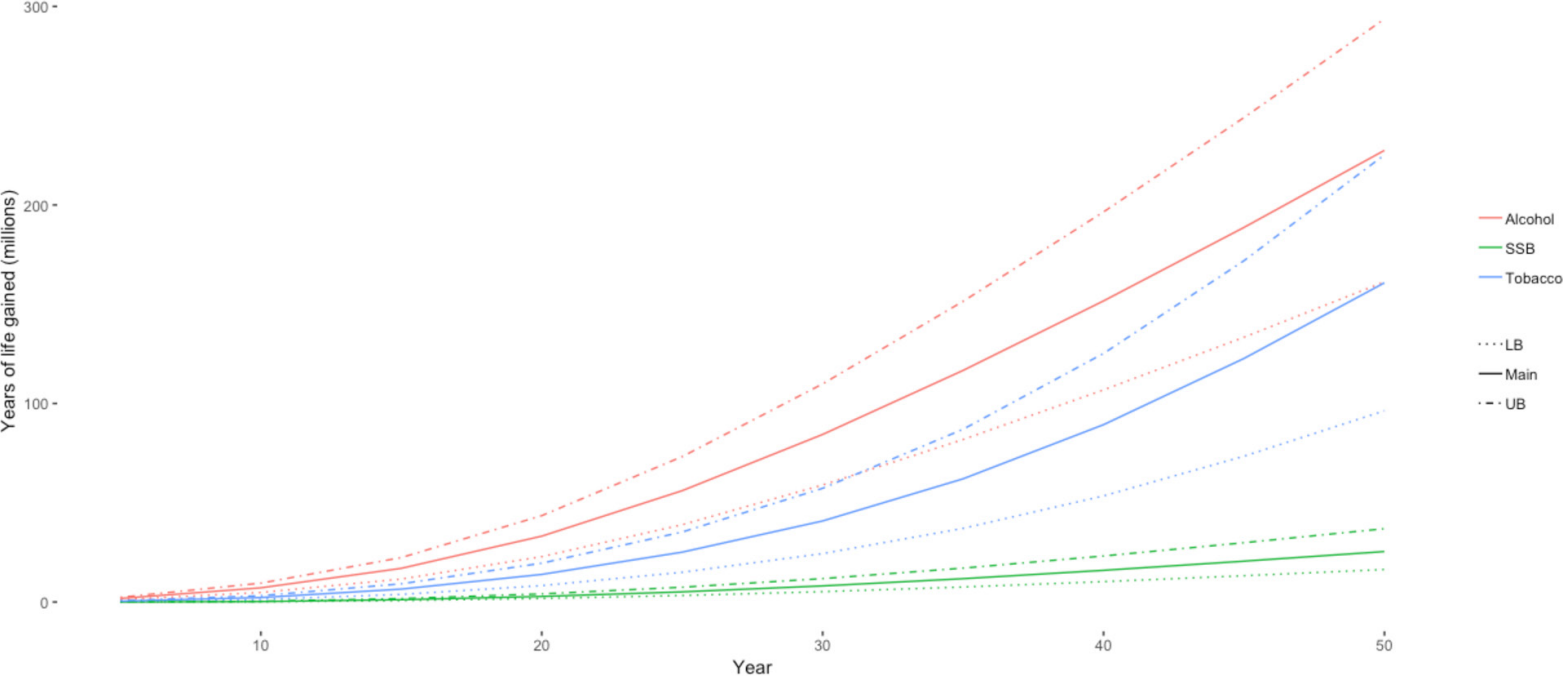
Trends in years of life gained from 20% price increase over 50 years. Source: authors’ estimates. LB, lower bound; SSB, sugar-sweetened beverages; UB, upper bound.

**Figure 4:**
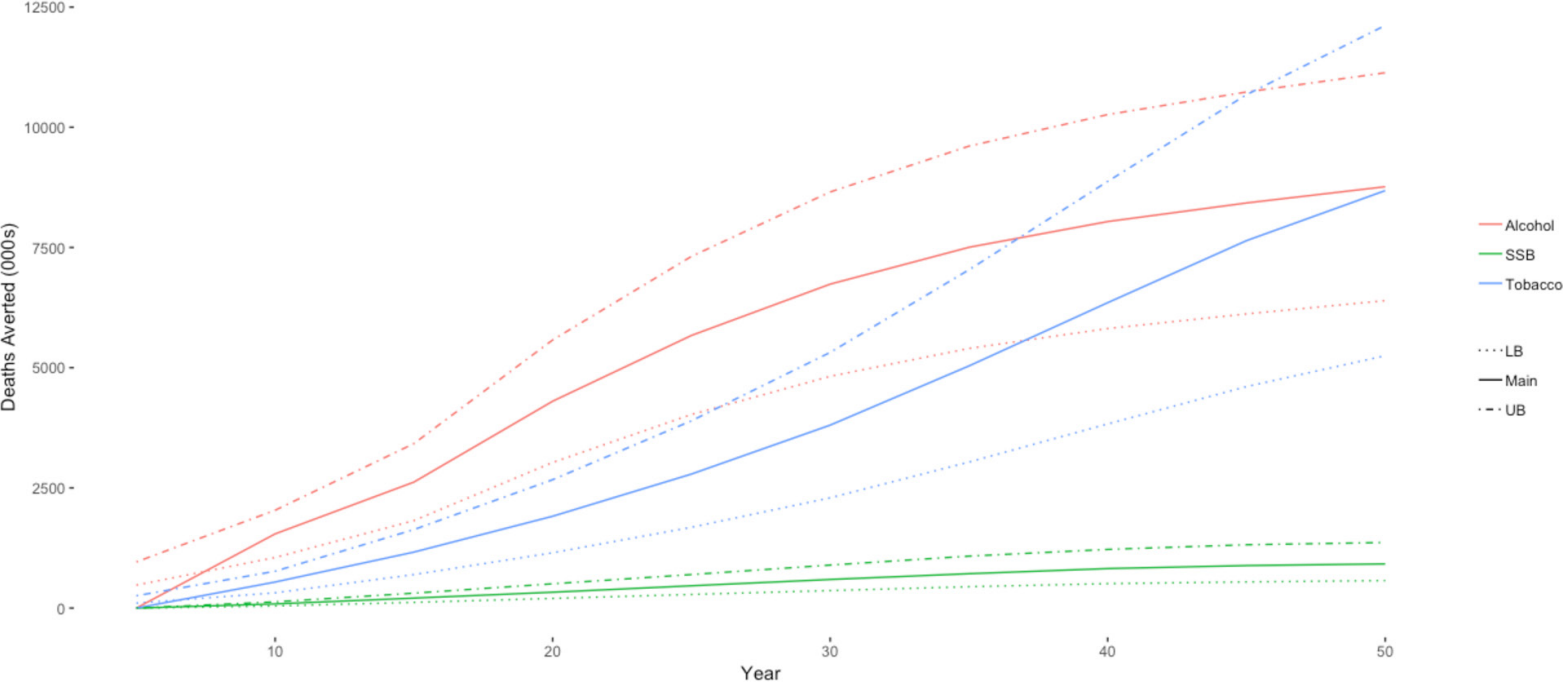
Trends in deaths averted from 20% price increase over 50 years. Source: authors’ estimates. LB, lower bound; SSB, sugar-sweetened beverages; UB, upper bound.

Figures 3 and 4 show how YLG and deaths averted accumulate over 50 years for all three commodities in the 20% price increase simulation. The accumulation of health gains is a function of the lag for each risk factor: it takes time for the change in consumption to improve health outcomes. For tobacco, the benefits from cessation accrue slowly and are largest for the younger age groups who do not begin smoking, since the benefits of cessation are greater for those who quit smoking (or do not start) at an earlier age.^28^ The benefits from tobacco taxation continue to accrue exponentially after the initial intervention because of the lower level of smoking for future youth cohorts in later years. For the alcohol taxation model, the effects start to accumulate faster than tobacco because of the instantaneous decrease in accidents and injuries: a reduction in consumption translates directly into a reduction in premature deaths. For SSBs, we assume that a new weight equilibrium is reached in 5 years, and health benefits accrue thereafter.

## Discussion

Our results demonstrate the potential for fiscal policy tools to reduce future global disease burden. The models estimate that, over a 50-year horizon, a one time tax increase that causes a 20% retail price increase would produce a gain of 161 million (UI: 96 to 225 million) life years if levied on tobacco, 227 million (UI: 161 to 294 million) life years if levied on alcoholic beverages, and 24 million (UI: 16 to 35 million) life years if levied on SSBs. Our results show that tobacco and alcohol tax increases, and more widespread SSB tax implementation can improve health benefits and increase government revenues.

Tobacco, alcohol and SSB consumption levels are increasing in some parts of the world, but fiscal policy tools to discourage consumption remain underused. In 2016, only 57 countries had tobacco taxes at WHO’s recommended level (70% tax share of the total retail price), and 51 countries had less than half the recommended rate.^37^ Taxes on alcohol tend to be lower than for tobacco, averaging less than 20% of retail price,^3^ while only a limited number of countries have imposed taxes on SSBs. Our results show that tobacco price increases and reduced consumption have the largest health effects in LIC and middle-income countries, reduced alcohol consumption in LMIC and HIC and reduced SSB consumption in HIC. However, all country income groups see substantial health and economic benefits. Furthermore, these benefits will increasingly go to LIC and middle-income countries due to their projected consumption growth. Globally, tobacco smoking prevalence is declining, but primarily in HIC. Many lower and middle-income countries are seeing no change in smoking prevalence, and a few are witnessing an increase.^37^ Similarly, even though alcohol consumption levels are decreasing in some regions, total alcohol consumption per capita is projected to increase globally in the next 10 years, driven by consumption increases in the South-East Asia and Western Pacific regions.^10^ Overweight and obesity levels are increasing in LIC and middle-income countries as countries progress through their nutrition transition,^7^ with concurrent increases in SSB consumption.^20^

Our simulations also suggest that a 20% price increase through taxes on tobacco, alcoholic beverages and SSBs would increase government revenues by US$1987 billion (UI: US$1613 to US$2297 billion), US$9428 (UI: US$8803 to US$10 053 billion) and US$724 billion (UI: US$680 to US$767 billion), respectively. These revenues can be earmarked for increased spending on tax enforcement or specific health and social programmes. Greater tax administration resources for detection equipment and enforcement teams can reduce tax avoidance,^38^ while increased funding for health and social programmes can be an important tool in swaying public opinion in favour of the tax increase—especially where perceptions have been negatively influenced by industry lobbyists. For example, governments could fund programmes to help consumers quit smoking, reduce alcohol consumption and/or choose healthy food alternatives. Poorer households have higher sensitivity to price changes and receive disproportionately larger benefits from reduced health expenditure^39 40^ and revenue-neutral programmes can be targeted at consumers who may bear the brunt of the tax, particularly poorer households. Finally, the tax revenues can defray less efficient and more distortive taxes, such as taxes on labour, to improve household outcomes.^41^

Our study has several limitations. First, our results may underestimate the effects of tax increases because we do not account for (1) morbidity effects or (2) the externality effects of consumption, such as secondhand smoke or drunk driving deaths. For smoking, alcohol consumption and SSB consumption, the global ratio of years lived with disability for every YLL is 0.15, 0.21 and 0.47, respectively, highlighting the large burden of morbidity for these risk factors.^1^

Second, as with all modelling studies, our simulations depend on the parameters used in our analysis. These are the best available estimates from the most recent scientific studies, but they identify past relationships between variables that may not apply going forward if lifestyles and environmental factors change. Third, our parameters on the relative risk of mortality for tobacco or alcohol represent an average for all age groups—they are not age or sex specific. However, the literature from which we derive our parameters includes all age groups and both sexes in its samples and adjusts estimates for age and sex.

Fourth, our consumption data were based on household and individual surveys. Recall bias and under-reporting by respondents in these surveys could result in underestimation of effect sizes. For example, a higher level of actual consumption would mean that we underestimated the total reduction in harmful consumption, thereby underestimating the potential health gains from a tax. A related issue is the difficulty of doing very large cross-country analyses. Where possible, we have employed country-specific data, including population distribution, mortality risks and consumption, but these data were not always available. Following other studies that have modelled non-communicable diseases,^14 15^ we have used a time horizon of 50 years because many of the health consequences of current tax policies are not observable for decades—for tobacco and alcohol, the full benefits of cessation are not realised 30 years and 20 years after consumption is reduced, respectively. The long time horizon requires assuming that preferences in demand and exogenous factors are static.

Fifth, we have not fully incorporated substitution effects between cigarettes and rustic tobacco such as bidis or with new forms of tobacco smoking such as e-cigarettes; between beer, wine, hard liquor and country liquor; and between SSBs and other beverages or foods. The data needed to support a model of these substitution effects for a global analysis are lacking. To partially account for substitution effects, in the alcohol model, we increased prices by the same level across all three beverage categories. The SSB model used an offset factor, whereby 50% of calories from SSBs were offset by other calories. If in fact taxes induce changes in the overall consumption of total grams of alcohol or total calories that are lower than estimated, then our health gains will be overestimated. For all three products, taxation on substitutes should also be considered to limit substitution after the tax—if such taxation is not applied, then our health gains may be overestimated.

We have also not modelled substitution to the illicit market due to a lack of data. Taking tobacco as an example, the relative size of the illicit market is unknown and reported figures show many inconsistencies.^42^ Furthermore, it has been found that the size of the illicit market does not increase due to tax increases.^38^ Black market size is primarily determined by the availability of sufficient tax administration and enforcement resources.^38^

Finally, we have not considered changes in health systems costs due to the projected increases in longevity. Increasing life expectancies may increase the burden on the healthcare system, and necessitate increased public health spending.^43^ With better data, future research should focus on addressing these limitations.

Fiscal policy tools accompanied with other government regulation can help reduce the prevalence of non-communicable diseases related to smoking, alcohol consumption and obesity. This paper shows the potential global gains from tobacco, alcohol and SSB taxation. Policy-makers should consider imposing or raising taxes within local tax structures to meet evidenced-based guidelines on taxing these commodities at levels that improve population health. Continuous monitoring, evaluation and research will help refine policies in the future to ensure they achieve maximum effectiveness.
