# Supplementary material for: The potential global gains in health and revenue from increased taxation of tobacco, alcohol and sugar-sweetened beverages: a modelling analysis
Source: BMJ Glob Health. 2020 Mar 29;5(3):e002143. doi: 10.1136/bmjgh-2019-002143 (PMC7170424; doi:10.1136/bmjgh-2019-002143)
Supplement: Supplementary data [file bmjgh-2019-002143supp001.pdf]

Supplementary Material

1 Modeling Overview

We simulated the health and economic effects of tobacco, alcohol, and sugar-sweetened beverage (SSB) taxation using separate mathematical models for each commodity that incorporated country level epidemiological, demographic, and consumption data. Model outputs included years of life gained (YLG), (premature) deaths averted, change in consumer spending, and change in tax revenue. Outcomes were aggregated and presented by World Bank country income group classifications: low income country (LIC), lower-middle income country (LMIC), upper-middle income country (UMIC), and high income country (HIC).

Figure A1 lays out the conceptual structure of the model. The tax is applied to the targeted product, which leads to a price increase and reduces consumption. Prices may change less than projected if the producer absorbs some or all of the costs of the tax. We use a common assumption[1–3] of a 100% pass-through of the tax. Reduced consumption changes the distribution of risk factors associated with the product within affected populations, ultimately affecting health outcomes. The magnitude of the change in consumption due to the price increase is determined by price elasticities of demand. The taxes also have direct economic consequences for consumer expenditures and for government receipts, as well as indirect outcomes that can include economic growth and labour outcomes.

We modeled two scenarios: a 20% and 50% price increase through tax increases. The models focused on health impacts of directly exposed individuals but did not consider externality effects of use, such as second-hand smoke or secondary impacts of alcohol and SSB consumption. The time horizon for the model was 50 years, with 2018 as the baseline year.

We conducted a sensitivity analysis using the Latin hypercube sampling method[4] on all estimates by drawing independent samples of parameters of elasticity and relative risk, varying them between 20% above and below their mean value using a

uniform distribution for each type of product for 1,000 iterations. We report the mean value of the resulting distribution as the point estimate, and the 2.5th percentile and 97.5th percentile are provided as a 95% uncertainty interval. Table A1 shows parameter values and Table A2 shows input sources. The next sections discuss methodological detail specific to the taxation modeling of the specific commodities, and estimation of global estimates.

The models used for each commodity followed a similar methodological approach, which is discussed in Sections 2 (health outcomes) and 3 (economic outcomes). The methodologies specific to the separate commodities are presented in Sections 4, 5 and 6. Approaches to estimating global effects are discussed in Section 7, and limitations are described in Section 8.

2 Health outcomes

2.1 Consumption

In the baseline scenario, future consumption per capita for each commodity for each country was estimated using published consumption projections; from Euromonitor[5] for SSBs, and from the World Health Organization (WHO) for alcohol[6] and tobacco[7]. Projections were not available for all countries; therefore, an average trend was calculated for each income group category and was applied to countries that were missing a trend. For SSBs, consumption trend projections were not available for any LICs. Hence, we used we used the estimated LMIC trend for LICs in our modeling. These trends, which were available for between five and fifteen years, were used to estimate a baseline consumption trajectory. We assumed that consumption growth was flat from year 15 through to year 50.

The effect of taxes on consumption were computed using price elasticities, as explained in the next section. In these computations, we assumed the entire tax was

Supplementary Material

Figure A1: Conceptual Framework of Modeling

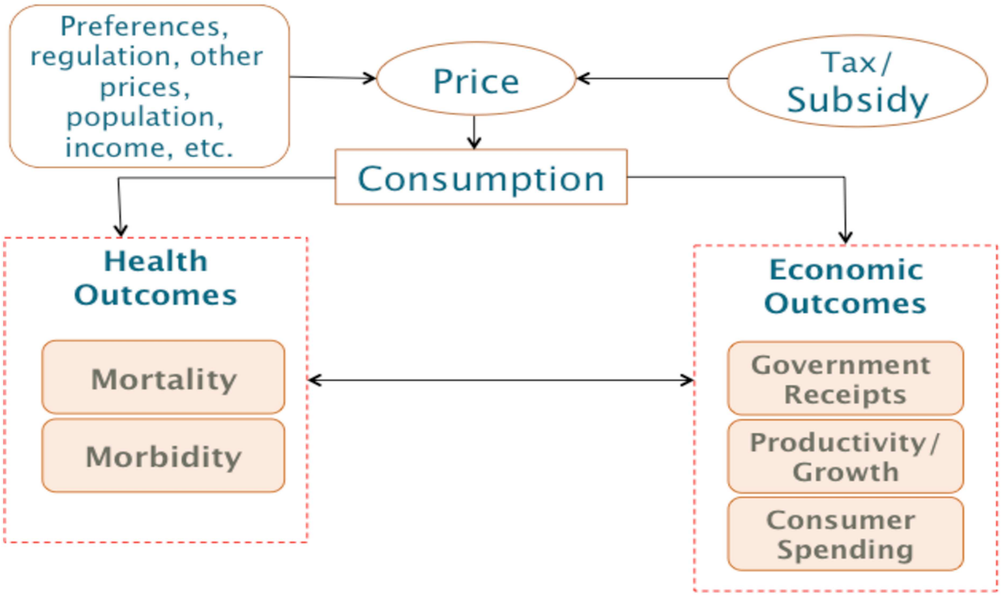

Arrow indicates direction of causation

Summan A, *et al. BMJ Global Health* 2020; 5:e002143. doi: 10.1136/bmjgh-2019-002143

Supplementary Material

Table A1: Model Parameters

Variable		*Data source*	*Value**
Tobacco			
Own-price elasticity	Cigarettes: LIC, LMIC, UMIC	Authors' assumptions based on IARC[8]	−0.5
	Cigarettes: HIC		−0.4
Relative risk of all-cause mortality	Cigarette smoker	Authors' assumptions based on multiple sources (See Table A3)	2.2
	Former smoker	Authors' assumption based on Doll and Peto[9]	See Figure 4 in Doll and Peto[9]
Alcohol			
Own-price elasticity	Alcohol: LIC, LMIC, UMIC	Authors' assumption based on Nelson[10]	−0.65
Relative risk of all-cause mortality	Daily consumption of grams of pure alcohol	Authors' estimates based on Grisworld *et al.*[11]	See Figure A2
Sugar-sweetened beverages			
Own-price elasticity	Sugar-sweetened beverages	Authors' estimates based on Cabrera Escobar *et al.*[12]	−1.2
Relative risk of all-cause mortality	Body mass index	Authors' estimates based on Aune *et al.*[13]	See Figure A3

LIC = low-income country; LMIC = lower-middle-income country; UMIC = upper-middle-income country; HIC = high-income country

Supplementary Material

Table 2: Input Sources

Input	*Source*
Baseline mortality rates	Global Burden of Disease[14]
Population	United Nations World Population Prospects[15]
Income groups	World Bank[16]
Tobacco	
Prices	WHO[17]
Tax rates	
Smoking prevalence and trends	WHO[7]
Smoking death rates	Global Burden of Disease[14]
Cigarette consumption	Euromonitor[18]; Ng *et al.*[19]
Alcohol	
Prices	WHO[20]; Euromonitor[5] ; OECD[21]
Tax rates	
Drinking prevalence and trends	WHO Global Health Observatory[20]
Grams of pure alcohol consumption	
Alcohol death rates	Global Burden of Disease[14]
Sugar-sweetened beverages	
Prices	Blecher *et al.*[22]; Euromonitor[5]
Consumption	Singh et al.[23]
Overweight and obese prevalence	Global Burden of Disease[24]
Height	NCD Risk Factor Collaboration[25]
Calorie consumption trends	Food and Agriculture Organization[26]

NCD = Non-communicable disease

passed on to consumers (i.e., full pass-through)[I]. Equation (Eq.) 1 shows how the level of consumption (disaggregated by sex, and by age where possible) was affected by changes in price:

$$C_{t,a,s} = (1 + \epsilon_a . \Delta p_{t-1}) . C_{t-1,a,s} \quad (1)$$

where $C_{t,a,s}$ is consumption of the good at time *t* for age group *a* and *s*ex *s*; ϵ_a is the age-specific own-price elasticity; and Δp_{t-1} is the change in the price of a good at time *t-1*. Elasticities quantify the effect of relative price changes on consumption of the taxed product. Five-year age intervals were used from ages 15 to 79, with the last age category consisting of individuals 80 years and older.

Own-price elasticities of demand identify how a change in price of a good leads to a proportionate change in consumption of the good. Cross-price elasticities quantify how a change in the price of one good leads to a proportionate change in the consumption of another good. Price elasticity can be defined as follows:

$$\epsilon_{x,p_y} = \frac{\frac{dq_x}{dp_y}}{\frac{q_x}{p_y}} \approx \frac{\frac{\Delta q_x}{q_x}}{\frac{\Delta p_y}{p_y}} \quad (2)$$

where p_y is the price of product y and q_x is the quantity of product x consumed. When $q_x = q_y$, the price elasticity is an own-price elasticity. We can infer changes in consumption for simulated price changes by rearranging the above equation as follows:

[I] The tobacco modeling literature[27] and the SSB modeling literature[28] commonly assumes a full pass-through of the tax. For alcohol, a recent cross-country study found, on average, overshifting of tax—increasing the price by an amount that is greater than the tax—with variations across beverage.[29] However, in practice the level of tax that is passed on to the consumer depends on demand and supply conditions and the competitive environment, which vary by country and across time. There is evidence of both undershifting and overshifting of taxes in different countries in the past, with many studies finding evidence for overshifting particularly for alcohol and tobacco.[29–33] Evidence for SSB taxes is comparatively limited.

$$\Delta q_x = q_x . \epsilon_{x,p_y} . \frac{\Delta p_y}{p_y} \quad (3)$$

We assumed a constant elasticity of demand function with the following functional form:

$$q(p) = \beta . p^{\alpha} \quad (4)$$

where p is price, and α is the elasticity and $\infty < \ \alpha < 0$.

2.2 Health Effects

Morbidity and mortality risks are associated with the consumption of alcohol, tobacco, and SSBs. We simulated the health effects of reduced consumption of these commodities using a standard, abridged baseline life table[ll] and estimated an intervention life table with modified mortality rates. Five-year age intervals were used from ages 15 to 79, with the last age category consisting of individuals 80 years and older. Individuals who reached the 80- to 84-year-old cohort were assumed to have a mortality rate of 1 and die at age 84. Alternatively, this can be considered as no policy effect for populations above age 80 and a focus on the relatively more productive years of life. Therefore, health effects were calculated for individuals between the ages of 30 and 80, and earlier deleterious effects of consumption were not realized until age 30. Following other modeling literature,[1] we assumed that cohorts born after 2018 were the same size as the current 0 to 5-year-old cohort.

3 Economic Outcomes

We calculated changes in consumer spending and tax revenue for each country over a 50-year period.

[ll] For a description on the construction of life tables, see United Nations(2009) or Gardner and Stewart(1966).

$$Averted\ Consumer\ Spending = \sum_{t=1}^{10}\sum_{a=1}^{14} \tilde{p}_t . \tilde{C}_{a,s,t}(\tilde{p}_t, y_t). \widetilde{pop}_{a,s,t} - \sum_{t=1}^{10}\sum_{a=1}^{14} p_t . C_{a,s,t}(p_t, y_t). pop_{a,s,t} \quad (5)$$

$$Tax\ Revenue = \sum_{t=1}^{10}\sum_{a=1}^{14} tax. \tilde{C}_{a,s,c,t}(\tilde{p}_{c,t}, y_t). \widetilde{pop}_{a,s,t} - \sum_{t=1}^{10}\sum_{a=1}^{14} tax. C_{a,s,c,t}(p_{c,t}, y_t). pop_{a,s,t} \quad (6)$$

where $\tilde{p}_t$ is the price after the tax, $\tilde{C}_{a,s,t}$ is the consumption level after the tax for age cohort a, sex s, and time t; and $\widetilde{pop}_{a,s,t}$ is the population of consumers after the tax. Consumption levels and patterns (prevalence) were calculated at the beginning of each 5-year period and assumed to hold for the defined period. All results are in 2018 USD, converted at current exchange rates. Future estimates of expenditures and revenues were discounted using a constant rate of 3%. The next sections discuss the methodological approaches specific to the modeling of the taxation of individual commodities.

4 Tobacco Taxation

4.1 Consumption

We focused on taxation of cigarettes, the most commonly consumed tobacco product, in our tobacco model — the average proportion of daily cigarette smoker prevalence to daily tobacco smoker prevalence across countries is 86%.[17]

The price change induced by a tax increase is assumed to reduce demand for cigarettes at both the extensive margin (number of smokers) and the intensive margin (number of cigarettes smoked by each smoker). Following the tobacco excise modeling literature,[1,3,8] we assumed the overall price elasticity of tobacco was split evenly between changes in the number of current smokers and changes in the intensity of consumption by continuing smokers. We also assumed that the elasticity

for younger groups, ages 15 to 25, is twice as large as for other age groups, in line with other studies.[1,3] Thus, the number of current smokers after the tax was given by the following:

$$\widetilde{CS_{a,s}} = \left(1 + \frac{1}{2}.\frac{\Delta p}{p}.\epsilon_a\right) CS_{a,s} \tag{7}$$

In the model, the number of current smokers decreased in response to a tax increase due to decreased initiation and increased cessation, increasing both the number of former and never smokers.

The reduction in the number of smokers at the beginning of the intervention was attributed entirely to cessation. Thus, the number of former smokers after the intervention was given as follows:

$$\widetilde{FS_{a,s}} = FS_{a,s} + \left|\frac{1}{2}.\frac{\Delta p}{p}.\epsilon_a.CS_{a,s}\right| \tag{8}$$

The number of never-smokers for future cohorts increased through reduced smoking initiation.

Based on previous literature, we assumed a price elasticity of –0.5 for all low- and middle-income countries, and –0.4 for HICs.[8] Data on smoking trends was taken from the WHO[7], who project trends in smoking prevalence by country from 2007 to 2025. We used these trends to project changes in prevalence for 15 years into the future. Data on the average daily number of cigarettes smoked were estimated using annual sales data from Euromonitor[5] and Ng et al.[19]. Sales data were preferable to survey data because of underreporting and recall bias in surveys.[7] For former smokers, we used data from the Global Adult Tobacco Survey, which are available for 39 countries.[36] We then calculated average former smokers as a proportion of current smokers by country income level and applied this ratio to countries for which former smoker data were missing. Price and tax data were also taken from the WHO; we

used prices for the most popular brand of cigarettes sold. Table A1 and A2 provide a complete list of parameter and input sources.

4.2 Health Effects

Following earlier literature,[2,37–40] we built on a commonly-used multistate life table modeling approach where we estimated separate life tables for smokers, never smokers, and current former smokers (smokers who quit smoking before the intervention) under a baseline scenario, and intervention life tables for never smokers, current smokers, current former smokers, and intervention former smokers (smokers who quit because of the intervention). The health effects in our model were attributable to changes in smoking status and not to changes in the intensity of smoking. The population of current former smokers did not change in the intervention scenario. The nonsmoker mortality rate was calculated as follows:

$$m_{a,s}^{NS} = m_{a,s} - \widetilde{m}_{a,s}^{\tilde{S}} \tag{9}$$

where $m_{a,s}^{NS}$ is the mortality rate for nonsmokers for age *a* and sex *s*, $\widetilde{m}_{a,s}^{\tilde{S}}$ is the mortality rate attributed to smoking, and $m_{a,s}$ is the mortality rate for the overall population. Mortality rates attributable to smoking were taken from the Global Burden of Disease (GBD) 2017 study[14]. We calculated the mortality rate for current smokers and intervention former smokers as follows:

$$m_{a,s}^{S} = m_{a,s}^{NS} \cdot RR^{S} \tag{10}$$

$$m_{a,s}^{FS} = m_{a,s}^{NS} \cdot RR_{C_{t,a}}^{FS} \tag{11}$$

where $m_{a,s}^{S}$ is the mortality rate for smokers, $m_{a,s}^{FS}$ is the mortality rate for former smokers, RR^{S} is the relative risk of mortality for smokers, and $RR_{C_{t,a}}^{FS}$ is the relative risk for former smokers who quit at time *t* and age *a*. These mortality rates were used to construct the baseline and intervention life tables. Successive age-sex cohorts were fed into the country-specific life table structures, and the number of

deaths and years of life lived were calculated in both the baseline and the intervention scenario over a 50-year period.

4.3 All-Cause Mortality and Smoking Behavior

A vast literature has addressed the relationship between mortality and smoking.[41–43] The British Doctors Study[44] was a landmark in this research; it followed one cohort of males for more than 50 years. Other meta-analyses have collated the data on this subject.[45–47] However, the variability across studies—on the length of follow-up with a cohort, the age groups studied, and the smoking behaviors included—makes comparisons challenging. To capture the complete potential effect of our interventions, we would like to use relative risks that apply to as many age groups as possible; that is, even though the accumulated effects of smoking are greatest in older smokers, heightened mortality rates have been observed among younger age groups as well. Table A3 highlights various studies on the effect of smoking on all-cause mortality. Based on these studies, we applied a relative mortality risk of 2.2 for current smokers for all individuals above age 30.

4.4 Health Benefits of Cessation

The risks of smoking-related disease decline as soon as an individual stops smoking. To account for the benefits of smoking cessation, we used relative risk estimates for former smokers from Doll and Peto's British Doctors study.[9] They followed a cohort of male doctors for more than 50 years and found that stopping smoking by age 30 avoids almost all of the excess risk of smoking, and stopping by age 50 avoids about half of it; on average, lifetime smokers lose 10 years of healthy life compared with lifelong nonsmokers. We calculated the relative risk of former smokers by age at quitting and determined the proportional reduction in risk of former smokers over time relative to current smokers based on the survival curves presented in Doll and Peto[9] of former smokers by their quit age over time relative to nonsmokers. Using the standardized reduction in risk relative to current smokers, we applied the proportional reduction in risk found in Doll and Peto[9] to our RR value of 2.2 for

current smokers, to arrive at a relative risk for former smokers over time. These values are summarized in Table A4.

Therefore, our model assumed that individuals who quit before age 35 return to a relative risk of a nonsmoker as soon as they quit, but smokers that quit after age 65 did not to see any reduction in their risk of mortality relative to current smokers. Other former smokers had a gradual reduction in risk, with those who quit at earlier ages having a greater reduction in mortality risk over the 50 years simulated in the model. Because of the difficulty in ascertaining the quit age of former smokers before the intervention, we assumed a relative risk of 1.4 for all former smokers before the intervention.

Supplementary Material

Table A3: Tobacco smoking and all-cause mortality

Country	All-cause mortality for smokers (reference: never smokers)	Smoking habit	Sex	Age	Source
China	1.26	current smoker and former smoker, >30 pack years	Male	40+	Gu *et al.*[48]
	1.38		Female	40+	
	2.42	current smoker	Male	35+	Lam[49]
	2.32		Female	35+	
United States	2.8	current smoker	Male	55+	Thun *et al.*[43]
	2.76		Female	55+	
	1.47	former smoker	Male	55+	
	1.45		Female	55+	
	1.18	current smoker, early smoking initiation before age 13 years	Both	30+	Choi and Stommel[50]
	1.19	former smoker, early smoking initiation before age 13 years	Both	30+	
	1.94	current smoker, 1-14 cigarettes /day	Female	34+	Dam *et al.*[51]
	2.32	current smoker, >15 cigarettes /day			
	1.52	former smoker			
	2.81	current smoker	Female	-	Kenfield[52]
Meta-Analysis (cross country)	1.83	current smoker	Both	60+	Gellert *et al.*[45]
	1.34	former smoker		60+	
	1.3	current smoker, 1-9 cigarettes/ day	Male	40+	Jacobs[53]
	1.8	current smoker, > 10 cigarettes/day			

Table A4: Relative risk of former smokers over time, by age of cessation

Quit age	Relative risk of mortality	
	2.5 years after cessation	Age 75–79
<35	1.00	1.00
35–44	1.50	1.06
45–54	1.67	1.14
55–64	1.75	1.29
65>	2.20	2.20

Source: Authors' estimates based Doll and Peto[9]

Supplementary Material

5 Alcohol Taxation

5.1 Consumption

We used alcohol consumption data on the grams of pure alcohol consumed daily by drinkers, trends in alcohol consumption, and the percentage of consumption attributed to each beverage category (beer, spirits, and wine) from WHO[6]. Consumption levels were disaggregated by sex but not by age group. When calculating economic effects, we ignored the "other" beverage category in our analysis, which was a median percentage share of pure alcohol consumption of 1%. WHO projects per capita alcohol consumption in grams of pure alcohol until 2030, but these changes are not attributed to changes in prevalence or intensity of consumption. We applied these projections to changes in the prevalence of drinkers from year 0 to 15 and assumed the same prevalence from year 16 to 50 in the baseline scenario. Following previous alcohol modeling,[54] changes in the price of alcohol resulting from increased taxation were modeled to affect drinking intensity, as described in equation (1).

We assumed a price elasticity of –0.64 for all countries according to a meta-analysis.[10] We did not model substitution across beverages because of the lack of consistent evidence on cross-price elasticities and the large number of countries in our analysis, which creates significant heterogeneity in relative beverage preferences, and therefore substitution. Instead, we simulated tax increases on each beverage which would lead to a uniform price increase across all three beverage categories: spirits, wine, and beer.

5.2 Health Effects

To estimate the health effects, we used a similar approach to that which was used for tobacco. We constructed separate life tables for drinkers and abstainers. We estimated mortality rates for non-drinkers as follows:

$$m^{A}_{a,s} = m_{a,s} - \widetilde{m}^{D}_{a,s} \qquad (12)$$

where $m^{A}_{a,s}$ is the death rate for abstainers by age *a* and sex *s*, and $\widetilde{m}^{D}_{a,s}$ is the death rate attributed to drinking by age and sex, which we obtained from the GBD study[14]. The mortality rate for drinkers was then estimated as follows:

$$m^{D}_{a,s} = m^{A}_{a,s} \cdot RR(g, t) \quad (13)$$

Where $m^{D}_{a,s}$ is the mortality rate for drinkers and $RR(g, t)$ is the relative risk of mortality, which is a function of time *t* since the intervention (period 1) and the level of daily alcohol consumption *g*. Relative risk estimates for the effect of alcohol consumption by average daily intake of grams of pure alcohol are from a recent meta-analysis[55] that accounted for potential bias in previous studies in the use of reference categories and classifications for drinkers. Figure A2 shows the relationship we estimated for alcohol relative risk of all-cause mortality and daily alcohol intake using this study.

5.3 Alcohol Health Lags

We accounted for the time lag between reduced alcohol consumption and the reduced risk of chronic alcohol-related diseases using estimates from Holmes *et al.*[56], who assessed the temporal relationship between alcohol consumption and harm for 23 chronic diseases.[III] We calculated a weighted average of the reduction in risk realized across chronic diseases after reducing alcohol consumption for 5-year intervals, weighting diseases by their respective contribution to total DALYs attributed to alcohol consumption[14]. We used this estimate to proxy for the mortality risk reduction over time from reduced consumption. Chronic disease accounts for only 79% of alcohol-related mortality, based on GBD estimates[14] of their contribution to alcohol-related harms. For injuries, which account for 21% of

[III] Although this is the most comprehensive analysis of the temporal relationship between alcohol consumption and harm, the authors were not able to quantify the time lag in reduction of risk from reduced consumption for all diseases, including tuberculosis.

Figure A2: Daily alcohol consumption and relative risk of all-cause mortality

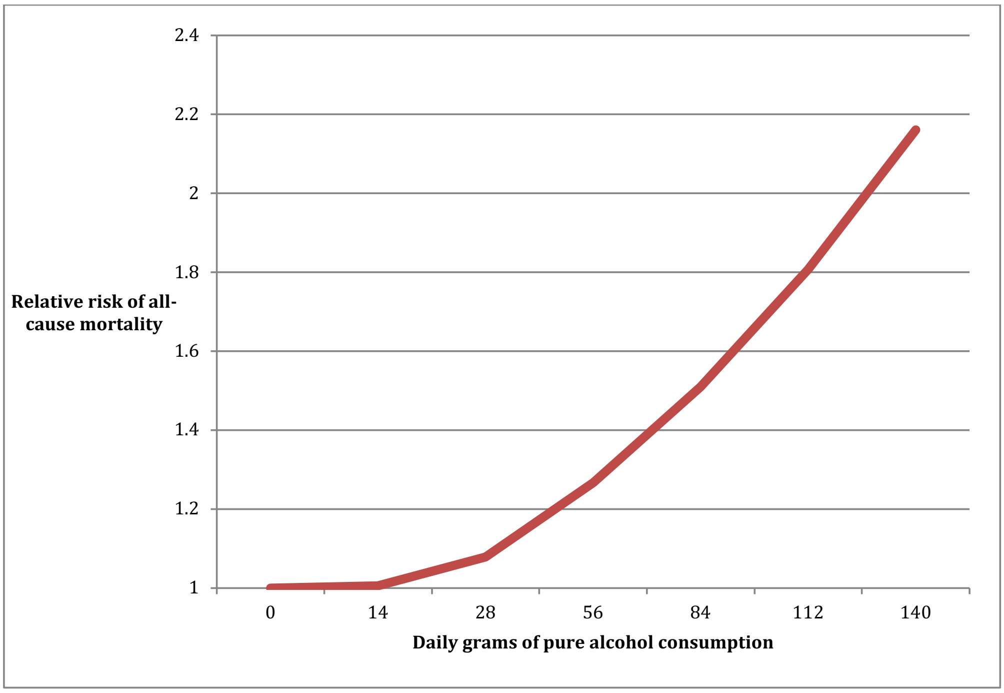

Relative risk curve estimated based on Stockwell *et al.*[55]

alcohol related mortality, we assumed an instantaneous reduction of risk from reduced alcohol consumption.

Using these estimates we assumed that an individual realizes the full effects from a reduction in alcohol consumption over several years; an individual who decreased consumption today will realize 50% of the full benefit in risk reduction in the next 5 years, 63% from years 5 to 10, 82% from years 10 to 15, 98% from years 15 to 20, and the full reduction in risk after 20 years. However, in the baseline consumption scenarios, which accounted for decreased drinking prevalence in trends, we assumed that all drinkers who stop drinking have the same risk as an abstainer as soon as they quit.

6 Sugar-Sweetened Beverage Taxation

6.1 Consumption

Data on baseline per capita SSB consumption by sex and age group were from the GBD study[57]. We projected future consumption of SSBs using Euromonitor regional forecasts of SSB consumption from 2017 to 2022. We used these 5-year trends and assumed they continue linearly 15 years into the future, and then assumed consumption growth remained flat until year 50. We simulated a one-time permanent reduction in consumption that arises from an increased price. The values of elasticities were based on the published literature[12], with an own-price elasticity of –1.20 .

A tax on SSBs may cause consumers to substitute other beverages, such as juice or milk, or even foods.[58] We did not explicitly include substitution to alternative beverages because of a lack of data on cross-price elasticities, but we assumed a 50% offset factor[IV] to account for substitution. Existing systematic reviews of cross-price elasticities have largely focused on the United States and may not be suitable

[IV] A 50% offset factor means that half the reduced consumption of SSB is being replaced by calories from other beverages or foods.

to our global analysis.[12] The use of a 50% offset factor is consistent with the levels of substitution found in other SSB tax modeling [59,60], where a 39% to 40% offset factor has been used. A full list of parameter values and input sources can be found in Table A1 and A2.

6.2 Health Effects

6.2.1 Body Mass Index Distributions

To model SSB taxation, we adopted an energy-balance approach to simulating shifts in body mass index (BMI) distribution associated with changes in beverage intake.[2,12,39] A previously estimated factor converting average energy imbalance to change in average body weight, 94kJ/kg, was used to simulate a change in average BMI for each age-sex group.[61] Data from the GBD study on the prevalence of obesity (BMI>30 kg/m^2) and obesity and overweight (BMI>25 kg/m^2) was used for all of the countries under consideration to construct baseline log-normal BMI distributions. We then re-simulated BMI distributions accounting for bodyweight changes arising from underlying country-specific trends in energy intakes as well as changes in energy intake that may result from the tax.[V] To account for changes in total calories under the baseline scenario, we incorporated estimates of the change in total calorie consumption from the Food and Agriculture Organization (FAO)[26], which projected total changes in calorie consumption by region until 2050.

We assumed that the tax does not affect individuals in low BMI categories. Therefore, any individual with a BMI of less than 24 fully offsets the decrease in SSB consumption due to the tax. To account for changes in calories and BMI under the baseline scenario, we incorporated estimates of the change in total calorie consumption from the FAO[26], who projected total changes in calorie consumption until 2050, separately for specific regions in developing countries and one aggregate

[V] Individuals with a BMI of less than 24 are assumed to fully offset the decrease in SSB consumption due to the tax.

projection for all developed countries. We assumed the trend in total calorie consumption between 2015 and 2050 continues linearly until 2067.

6.2.2 Simulating Baseline Body Mass Index

Data from the GBD study on the prevalence of obesity (BMI>30 kg/m^2) and obesity and overweight (BMI>25 kg/m^2) was used for all of the countries under consideration to construct baseline log-normal BMI distributions. We assumed that underlying these prevalence estimates is a log-normal distribution. Log-normal distributions are right-skewed, have a domain that is the positive real number line, and are empirically found to provide a good fit for observed BMI distributions [62]. We simulated baseline BMI distributions in the initial period, which then served as the inputs to simulate subsequent BMI distributions under the baseline scenario, as well as to simulate initial BMI distributions under the intervention scenario. The log-normal cumulative distribution function is given by the following functional form:

$$F(x) = \Phi\left(\frac{\ln(x) - \mu}{\sigma}\right) \tag{14}$$

where μ and σ are parameters mean and standard deviation, respectively, of the distribution, and Φ is the standard normal cumulative distribution function.

For each country-age-sex group we have both an estimate of obesity prevalence, $\hat{p}_{ob}$, which assuming a log-normal distribution is equivalent to $1 - F(30)$, and obesity and overweight prevalence, $\hat{p}_{ow}$, which is equivalent to $1 - F(25)$ from the GBD study. This allowed us to construct a system of two simultaneous equations that, when solved, allowed us to identify the parameters of the log-normal distribution, μ and σ, which resulted in $\hat{p}_{ob}$ and $\hat{p}_{ow}$. These simultaneous equations are as follows:

$$\Phi^{-1}(1-\hat{p}_{ob}) = \frac{\ln(30)-\hat{\mu}}{\hat{\sigma}} \quad (15)$$

$$\Phi^{-1}(1-\hat{p}_{ow}) = \frac{\ln(25)-\hat{\mu}}{\hat{\sigma}} \quad (16)$$

Jointly solving these allowed us to parameterize BMI distributions in the initial period of the baseline scenario.

6.2.3 Simulating Shifts in Body Mass Index Distributions

For a given change in daily beverage consumption (and therefore daily energy intake), we simulated a change in the mean of the BMI distribution as follows:

$$\Delta meanBMI_{a,c,s,t+1} = \frac{\Delta Energy_{a,c,s,t}\ 94\,kJ/kg}{{height_{a,c,s,t}}^{2}} \quad (17)$$

where:

$$\Delta Energy_{a,c,s,t+1} = (C_{a,s,c,t+1}(p_{t+1}, y_{t+1}) - C_{a,s,c,t}(p_t, y_t))\ SSBEnergyContent \quad (18)$$

This allowed us to construct a new mean:

$$meanBMI_{a,c,s,t} = meanBMI_{a,c,s,t-1} + \Delta meanBMI_{a,c,s,t} \quad (19)$$

We assumed a shift in mean BMI also resulted in a change in the standard deviation of the BMI distribution. Ideally, one would use the joint distribution of SSB intake and BMI to simulate the effect of changes in SSB intake on BMI; however, these data did not exist and we therefore made a numerical assumption derived from the

observed relationship between proportionate shifts in mean BMI and its relationship to BMI standard deviation. This relationship is as follows:

$$sdBMI_{a,c,s,t} = \left(\frac{meanBMI_{a,c,s,t} - meanBMI_{a,c,s,t-1}}{meanBMI_{a,c,s,t}}\right) sdBMI_{a,c,s,t-1}1.18 \quad (20)$$

Based on the simulated mean and standard deviation, we calculated the μ and σ that would characterize the lognormal distribution with that mean and standard deviation. This process was repeated iteratively, providing a trajectory of μ and σ under the baseline and intervention scenarios. This trajectory allowed us to calculate BMI category prevalence for each age-sex-country group under both the baseline and intervention scenarios.

6.2.4 Estimating Health Effects

The previous section describes how we simulated the trajectory of BMI distributions for each age-sex-country population, under a baseline scenario and an intervention scenario. We used a potential impact fraction (PIF) to translate these shifts in BMI distribution into changes in mortality rates. The general PIF is given as follows:

$$PIF_{a,s,c,t} = \frac{\sum_{i=1}^{n} q_{i,a,s,c,t}.RR_i - \sum_{i=1}^{n} p_{i,a,s,c,t-1}.RR_i}{\sum_{i=1}^{n} p_{i,a,s,c,t-1}.RR_i} \quad (21)$$

where, for BMI estimation, RR_i is the relative risk of all-cause mortality in BMI distribution category i, $p_{i,a,s,c,t-1}$ is the prevalence of BMI category i, and, $q_{i,a,s,c,t}$ is the simulated BMI category prevalence arising from changes in SSB intake. For our model we assigned individuals to eight BMI categories. These were in turn used to scale the prevailing mortality rate to estimate an intervention mortality rate:

$$\tilde{m}_{a,s,c,t} = (1 + PIF_{a,s,c,t}).m_{a,s,c,t-1} \quad (22)$$

The resulting simulated mortality rates were fed into a standard life table structure.

For BMI, we obtained relative risks from a meta-analysis[13] estimating the all-cause mortality risk associated with different levels of BMI, from which we estimated a quadratic equation. We did not model the changes in disease incidences explicitly and used the relationship between overall mortality and BMI to model health effects which implicitly accounted for all the BMI-related diseases: stroke, ischemic heart disease, hypertensive heart disease, diabetes mellitus, osteoarthritis, postmenopausal breast cancer, colon cancer, endometrial cancer, and kidney cancer.[63] Figure A3 shows the estimated relationship between BMI and all-cause mortality we use based on Aune *et al*.[64].

Unlike the alcohol and tobacco models, there were few data on the possible lags in the decrease in risk factor exposure and the corresponding health benefits. Therefore, for our analysis we assumed a lag of 5 years, where the full benefits of reduced SSB consumption began to accrue 5 years after the tax is implemented. This can also be thought of as the time it takes for an individual to reach a new equilibrium weight after reducing calories because of the intervention.

Figure A3: Body-mass index and relative risk of all-cause mortality

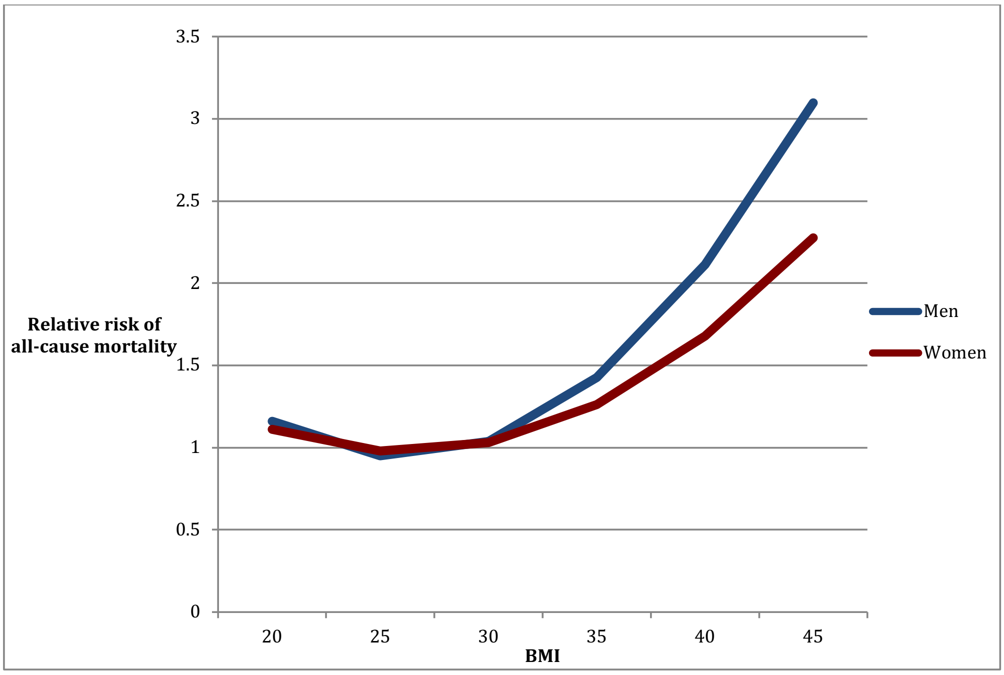

Relative risk curve estimated based on Aune *et al.*[64]

7 Estimating Global Effects

To estimate global health effects, we estimated the total health effects per 100,000 individuals by each country income group, using the four World Bank income group classifications.[65]I We then matched countries not covered in our sample by income level to these estimates and imputed the in-sample, weighted health effects to these countries. Parameter and input data were available for countries across all three commodities for estimating health effects: tobacco data was available for countries comprising 92% of the global population; alcohol data, 97%; and SSBs, 95%. To estimate global economic effects, we first matched countries by exposure level (tertiles of smoking prevalence for tobacco, daily alcohol consumption for alcohol, and daily SSB consumption for SSBs), region, and income, and imputed in-sample average missing economic parameter data (price and tax) for countries that were missing these data, but had underlying consumption pattern data, and simulated economic effects for these countries. For countries missing both economic and consumption data, we imputed the population-weighted average economic effects

calculated at the country's income level. Tobacco, alcohol, and SSB economic parameter data were available for countries representing 91%, 43%, and 83% of the global population, respectively. For alcohol our sample covered 65% and 40% of the global population for consumer expenditure and tax revenue estimates, respectively. The countries included in the sample for different models are listed in Table A5. The availability of data for a large number of countries decreases the potential error from extrapolation to the global level. For SSBs and tobacco we did not have data for countries representing the 9% of the global population in the LIC group for economic outcome estimates; therefore, for extrapolation to the low-income group, we used LMIC estimates.[VI]

Our sample estimates could have been extrapolated to the global level in many ways, including by region, income level, level of exposure, tax rates, prices, or a combination of these factors. We chose income level, for a number of reasons. Income level is highly associated with life expectancy, population distribution, and overall health patterns across countries. These factors enter our models through life tables in the form of mortality rates across different cohorts over time. For health effects, evidence exists for an association between income level and alcohol consumption,[66] income and tobacco prevalence,[67] and income and obesity.[68] Similarly, for economic outcomes, consumption levels and patterns, and baseline tax rates and prices are parameters associated with income level across countries.

8 Limitations

Our analysis had several limitations. First, our results potentially underestimated the effects of tax increases, for the following reasons:

a) We focused only on mortality. In ignoring morbidity effects, our results underestimated the total averted disease burden. For smoking, alcohol consumption, and SSB consumption, the GBD study estimates[14] a global ratio

[VI] We also estimated effects for low-income countries using estimates from countries in the lower-middle-income group that represent the bottom 50% of income; we found no significant difference in estimates.

of years lived with disability (YLD) for every years of life lost (YLL) of 0.15, 0.21, and 0.47, respectively, highlighting the large burden of morbidity for these risk factors.

b) We did not account for externality effects of consumption, such as second-hand smoke or drunk driving deaths where the driver injures other individuals. These effects, relative to the direct health effect, are the subject of debate. Second-hand smoke may have significant side-effects, but with the spread of bans on smoking in workplaces and public places, tobacco health effects may be increasingly concentrated on the individual smoker. Crashes related to drink driving are a serious cause of injury, but a very small proportion of drinkers account for a very large proportion of these effects. Therefore, taxing all drinkers may be less efficient than focusing on regulatory and technological barriers to driving drunk. Other effects, including alcohol consumption's association with unsafe sex and sexually transmitted infections, should be part of future attempts to derive more comprehensive estimates than ours.

c) For the SSB model, we focused only on BMI-related mortality similar to other models in the literature[2,63]; we did not include the direct effect of SSB consumption on diabetes (independent of BMI), but only the effect that occurs through changing BMI.

Second, our relative risk of mortality parameters represent an average for all age groups and were not age- or sex-specific for alcohol and tobacco. However, the literature from which we derived our parameters included these age groups and both sexes in their samples and adjusted estimates for age and sex. For alcohol, relative risks of mortality parameters we used came from a recent meta-analysis[55] that took into account previous study designs that may have biased results toward higher protective effects for low levels of alcohol consumption that are important to consider, but does not provide separate estimates by sex.

Third, as with all modeling studies, our simulations are dependent on the parameters used in our analysis. These are the best available estimates from the

most recent scientific studies but identify past relationships between variables that may not apply going forward due to other secular changes in lifestyle and environmental factors. Our consumption data for many interventions are based on household and individual surveys, which may not capture true consumption patterns, given recall bias and underreporting. To circumvent this issue, we tried to use sales data whenever possible. A related issue is the difficulty of doing very large cross-country analyses. Where possible we have employed country-specific data, including population distribution, mortality risks, and consumption, but this data was not always available. Our parameters were also taken from meta-analyses. Our health analyses were able to cover more than 92% of the global population in our sample. However, economic analyses covered fewer countries because of the lack of data. The gaps in data were greatest for alcohol—where tax rates and prices across three beverage categories were required—increasing the likelihood of error when results are extrapolated to the global level.

Fourth, we have not extensively modeled substitution effects because we lack data on appropriate parameters. For alcohol we simply increased prices by the same level across all three beverage categories and used within product substitution where we simulated price increases by beverage per serving. The SSB model uses an offset factor, where 50% of calories are offset by other calories. Therefore, our results may be overestimated if there is unaccounted-for substitution in the model. For SSBs, for example, the level of substitution may be higher due to non-beverage substitution effects in increasing overall calorie intake. [69] For alcohol, conversely, we may have underestimated the total benefits of the tax because reduced consumption of harmful complements due to the tax increase, such as tobacco, was ignored.[70]

Finally, our analysis was conducted over a long-time horizon. Although this period is often used in the modelling literature, it challenges our analytical abilities because of the potential for changing preferences in demand and other important exogenous factors.

Supplementary Material

Table A5: Countries included in analyses

Country	Alcohol			Sugar-sweetened beverages		Tobacco	
	Health	Economic: expenditure	Economic: tax	Health	Economic	Health	Economic
Afghanistan	Yes			Yes			
Albania	Yes	Yes	Yes	Yes		Yes	
Algeria	Yes	Yes	Yes	Yes	Yes	Yes	Yes
Angola	Yes	Yes	Yes	Yes			
Antigua and Barbuda	Yes			Yes			
Argentina	Yes	Yes	Yes	Yes	Yes	Yes	Yes
Armenia	Yes	Yes	Yes	Yes		Yes	Yes
Australia	Yes	Yes	Yes	Yes	Yes	Yes	Yes
Austria				Yes	Yes	Yes	Yes
Azerbaijan	Yes	Yes	Yes	Yes	Yes	Yes	Yes
Bahamas	Yes	Yes		Yes		Yes	
Bahrain	Yes			Yes	Yes	Yes	Yes
Bangladesh	Yes	Yes		Yes	Yes	Yes	Yes
Barbados	Yes			Yes		Yes	Yes
Belarus	Yes	Yes	Yes	Yes		Yes	Yes
Belgium	Yes			Yes	Yes	Yes	Yes
Belize	Yes	Yes	Yes	Yes			
Benin	Yes	Yes	Yes	Yes		Yes	Yes
Bhutan	Yes			Yes			
Bolivia	Yes			Yes			
Bosnia and Herzegovina	Yes	Yes		Yes	Yes	Yes	Yes
Botswana	Yes			Yes		Yes	Yes
Brazil	Yes	Yes	Yes	Yes	Yes	Yes	Yes
Brunei Darussalam	Yes			Yes		Yes	
Bulgaria				Yes	Yes	Yes	Yes
Burkina Faso	Yes			Yes		Yes	Yes
Burundi	Yes			Yes			
Cabo Verde	Yes	Yes	Yes	Yes		Yes	Yes

Cambodia	Yes			Yes	Yes	Yes	Yes
Cameroon	Yes	Yes	Yes	Yes	Yes		
Canada	Yes	Yes	Yes	Yes	Yes	Yes	Yes
Central African Republic	Yes			Yes			
Chad	Yes	Yes		Yes			
Chile	Yes	Yes	Yes	Yes	Yes	Yes	Yes
China	Yes	Yes	Yes	Yes	Yes	Yes	Yes
Colombia	Yes			Yes	Yes	Yes	Yes
Comoros	Yes			Yes		Yes	Yes
Congo	Yes			Yes		Yes	Yes
Costa Rica	Yes	Yes	Yes	Yes		Yes	Yes
Cote d'Ivoire	Yes			Yes	Yes	Yes	Yes
Croatia	Yes	Yes	Yes	Yes		Yes	Yes
Cuba	Yes			Yes		Yes	Yes
Cyprus	Yes			Yes		Yes	Yes
Czech Republic	Yes			Yes	Yes	Yes	
Dem. Republic of the Congo	Yes						
Denmark	Yes	Yes	Yes	Yes	Yes	Yes	Yes
Djibouti	Yes			Yes		Yes	Yes
Dominican Republic				Yes	Yes	Yes	Yes
Ecuador	Yes			Yes	Yes	Yes	Yes
Egypt	Yes			Yes	Yes	Yes	Yes
El Salvador	Yes	Yes	Yes	Yes		Yes	Yes
Equatorial Guinea	Yes			Yes			
Eritrea						Yes	Yes
Estonia				Yes	Yes	Yes	Yes
Ethiopia	Yes	Yes	Yes	Yes		Yes	Yes
Fiji	Yes			Yes		Yes	Yes
Finland	Yes	Yes	Yes	Yes	Yes	Yes	Yes
France	Yes			Yes	Yes	Yes	Yes
Gabon	Yes			Yes			
Gambia	Yes	Yes	Yes	Yes		Yes	
Georgia	Yes			Yes	Yes	Yes	Yes
Germany	Yes			Yes	Yes	Yes	Yes

Supplementary Material

Ghana	Yes	Yes	Yes	Yes		Yes	Yes
Greece	Yes	Yes	Yes	Yes	Yes	Yes	Yes
Grenada	Yes	Yes		Yes			
Guatemala	Yes	Yes	Yes	Yes	Yes		
Guinea	Yes			Yes			
Guinea-Bissau	Yes			Yes			
Guyana				Yes			
Haiti	Yes			Yes		Yes	Yes
Honduras	Yes			Yes			
Hungary	Yes			Yes	Yes	Yes	Yes
Iceland	Yes			Yes	Yes	Yes	Yes
India	Yes	Yes		Yes	Yes	Yes	Yes
Indonesia	Yes			Yes	Yes	Yes	Yes
Iran (Islamic Republic of)	Yes			Yes	Yes	Yes	Yes
Iraq	Yes			Yes			
Ireland	Yes			Yes	Yes	Yes	Yes
Israel	Yes			Yes	Yes	Yes	Yes
Italy	Yes			Yes	Yes	Yes	Yes
Jamaica	Yes	Yes		Yes		Yes	Yes
Japan	Yes			Yes	Yes	Yes	Yes
Jordan	Yes			Yes	Yes		
Kazakhstan	Yes			Yes	Yes	Yes	Yes
Kenya	Yes	Yes		Yes	Yes	Yes	Yes
Kiribati	Yes			Yes		Yes	Yes
Kuwait	Yes			Yes	Yes	Yes	Yes
Kyrgyzstan	Yes	Yes	Yes	Yes		Yes	Yes
Lao People's Dem. Republic	Yes			Yes		Yes	Yes
Latvia				Yes	Yes	Yes	Yes
Lebanon	Yes			Yes		Yes	Yes
Lesotho	Yes			Yes		Yes	
Liberia	Yes	Yes	Yes	Yes		Yes	Yes
Libya	Yes			Yes			
Lithuania	Yes	Yes	Yes	Yes		Yes	Yes

Supplementary Material

Luxembourg	Yes			Yes	Yes	Yes	Yes
Madagascar	Yes			Yes			
Malawi	Yes	Yes	Yes	Yes		Yes	
Malaysia	Yes	Yes	Yes	Yes	Yes	Yes	Yes
Maldives	Yes			Yes		Yes	Yes
Mali	Yes			Yes		Yes	Yes
Malta	Yes	Yes	Yes	Yes		Yes	Yes
Mauritania	Yes			Yes			
Mauritius	Yes	Yes		Yes		Yes	Yes
Mexico	Yes	Yes	Yes	Yes	Yes	Yes	Yes
Micronesia	Yes			Yes			
Mongolia	Yes	Yes	Yes	Yes		Yes	Yes
Montenegro				Yes		Yes	Yes
Morocco	Yes			Yes	Yes	Yes	Yes
Mozambique	Yes	Yes	Yes	Yes		Yes	Yes
Myanmar	Yes	Yes	Yes	Yes		Yes	Yes
Namibia	Yes			Yes		Yes	Yes
Nepal	Yes	Yes	Yes	Yes		Yes	Yes
Netherlands	Yes			Yes	Yes	Yes	Yes
New Zealand	Yes			Yes	Yes	Yes	Yes
Nicaragua	Yes	Yes	Yes	Yes			
Niger	Yes	Yes	Yes	Yes		Yes	Yes
Nigeria	Yes	Yes	Yes	Yes	Yes	Yes	Yes
Norway	Yes	Yes	Yes	Yes	Yes	Yes	Yes
Oman	Yes			Yes	Yes	Yes	Yes
Pakistan	Yes			Yes	Yes	Yes	Yes
Panama	Yes	Yes		Yes	Yes	Yes	Yes
Papua New Guinea	Yes			Yes	Yes	Yes	Yes
Paraguay	Yes	Yes	Yes	Yes	Yes	Yes	Yes
Peru	Yes	Yes		Yes	Yes		
Philippines	Yes			Yes	Yes	Yes	Yes
Poland	Yes	Yes	Yes	Yes	Yes	Yes	Yes
Portugal	Yes	Yes	Yes	Yes	Yes	Yes	Yes
Qatar	Yes			Yes	Yes	Yes	Yes

Summan A, *et al. BMJ Global Health* 2020; 5:e002143. doi: 10.1136/bmjgh-2019-002143

Republic of Korea	Yes					Yes	Yes
Republic of Moldova	Yes	Yes		Yes		Yes	Yes
Romania	Yes	Yes	Yes	Yes	Yes	Yes	Yes
Russian Federation	Yes	Yes				Yes	Yes
Rwanda	Yes	Yes	Yes	Yes		Yes	Yes
Saint Lucia	Yes	Yes		Yes			
Samoa				Yes		Yes	Yes
Sao Tome and Principe	Yes	Yes	Yes	Yes			
Saudi Arabia	Yes			Yes	Yes	Yes	Yes
Senegal	Yes	Yes	Yes	Yes		Yes	Yes
Serbia	Yes	Yes	Yes	Yes	Yes	Yes	Yes
Seychelles	Yes	Yes		Yes		Yes	Yes
Sierra Leone	Yes	Yes	Yes	Yes		Yes	Yes
Singapore	Yes			Yes	Yes	Yes	Yes
Slovakia	Yes			Yes	Yes	Yes	Yes
Slovenia	Yes	Yes	Yes	Yes		Yes	Yes
Solomon Islands	Yes			Yes			
Somalia	Yes			Yes			
South Africa	Yes	Yes	Yes	Yes	Yes	Yes	Yes
Spain	Yes	Yes	Yes	Yes	Yes	Yes	Yes
Sri Lanka	Yes	Yes	Yes	Yes	Yes	Yes	Yes
St. Vincent and the Grenadines	Yes			Yes			
Suriname	Yes	Yes		Yes		Yes	Yes
Swaziland	Yes			Yes		Yes	Yes
Sweden	Yes	Yes	Yes	Yes	Yes	Yes	Yes
Switzerland	Yes	Yes	Yes	Yes	Yes	Yes	Yes
Syrian Arab Republic	Yes	Yes		Yes			
TFYR Macedonia	Yes	Yes	Yes	Yes	Yes		

Supplementary Material

Tajikistan				Yes			
Thailand	Yes	Yes	Yes	Yes	Yes	Yes	Yes
Timor-Leste	Yes	Yes		Yes			
Togo				Yes		Yes	Yes
Tonga	Yes			Yes		Yes	Yes
Trinidad and Tobago	Yes	Yes	Yes	Yes			
Tunisia	Yes			Yes	Yes	Yes	Yes
Turkey	Yes	Yes	Yes	Yes	Yes	Yes	Yes
Turkmenistan	Yes			Yes			
Uganda	Yes	Yes		Yes		Yes	Yes
Ukraine	Yes	Yes		Yes	Yes	Yes	Yes
United Arab Emirates	Yes			Yes	Yes	Yes	Yes
United Kingdom	Yes			Yes	Yes	Yes	Yes
United Republic of Tanzania	Yes			Yes		Yes	Yes
United States of America	Yes			Yes	Yes	Yes	Yes
Uruguay	Yes	Yes	Yes	Yes	Yes	Yes	Yes
Uzbekistan	Yes			Yes	Yes	Yes	Yes
Vanuatu	Yes			Yes		Yes	Yes
Venezuela (Bolivarian Republic of)	Yes	Yes	Yes	Yes	Yes		
Viet Nam	Yes			Yes	Yes	Yes	Yes
Yemen	Yes			Yes		Yes	Yes
Zambia	Yes	Yes	Yes	Yes		Yes	Yes
Zimbabwe	Yes					Yes	Yes

Summan A, *et al. BMJ Global Health* 2020; 5:e002143. doi: 10.1136/bmjgh-2019-002143

9 References

1 Verguet S, Gauvreau CL, Mishra S, *et al.* The consequences of tobacco tax on household health and finances in rich and poor smokers in China: an extended cost-effectiveness analysis. *Lancet Glob Health* 2015; **3**: e206–16.

2 Manyema M, Veerman LJ, Chola L, *et al.* The Potential Impact of a 20% Tax on Sugar-Sweetened Beverages on Obesity in South African Adults: A Mathematical Model. *PLoS ONE* 2014; **9**: e105287.

3 Jha P, Joseph R, Li D. Tobacco taxes: a win-win measure for fiscal space and health. 2012.

4 Helton JC, Davis FJ, Johnson JD. A comparison of uncertainty and sensitivity analysis results obtained with random and Latin hypercube sampling. *Reliab Eng Syst Saf* 2005; **89**: 305–30.

5 Euromonitor International. Euromonitor International. 2018.

6 WHO. Global Health Observatory data repository. 2016. http://apps.who.int/gho/data/node.home (accessed May 6, 2018).

7 WHO. WHO global report on trends in prevalence of tobacco smoking 2015. 2015.

8 IARC. Effectiveness of tax and price policies for tobacco control. 2011; **14**. DOI:10.1136/tc.2010.039982.

9 Doll R, Peto R, Boreham J, Sutherland I. Mortality in relation to smoking: 50 years' observations on male British doctors. *BMJ* 2004; **328**: 1519.

10 Nelson JP. Meta-analysis of alcohol price and income elasticities – with corrections for publication bias. *Health Econ Rev* 2013; **3**: 17.

11 Stockwell T, Zhao J, Greenfield T, Li J, Livingston M, Meng Y. Estimating under- and over-reporting of drinking in national surveys of alcohol consumption: identification of consistent biases across four English-speaking countries. *Addiction* 2016; **111**: 1203–13.

12 Cabrera Escobar M a, Veerman JL, Tollman SM, Bertram MY, Hofman KJ. Evidence that a tax on sugar sweetened beverages reduces the obesity rate: a meta-analysis. *BMC Public Health* 2013; **13**: 1072.

13 Aune D, Sen A, Prasad M, *et al.* BMI and all cause mortality: systematic review and non-linear dose-response meta-analysis of 230 cohort studies with 3.74 million deaths among 30.3 million participants. *BMJ* 2016; **9**: i2156.

14 Institute for Health Metrics and Evaluation. Global Burden of Disease Study 2017 (GBD 2017) Data Resources. 2019. http://ghdx.healthdata.org/gbd-2017.

Supplementary Material

15 World Population Prospects - Population Division - United Nations. https://population.un.org/wpp/ (accessed Nov 8, 2019).

16 New country classifications by income level: 2016-2017. World Bank Blogs. https://blogs.worldbank.org/opendata/new-country-classifications-2016 (accessed Nov 8, 2019).

17 World Health Organization. WHO Report on the Global Tobacco Epidemic, 2019. Geneva, 2019.

18 Euromonitor International. Euromonitor International. 2018.

19 Ng M, Freeman MK, Fleming TD, *et al.* Smoking Prevalence and Cigarette Consumption in 187 Countries, 1980-2012. *JAMA* 2014; **311**: 183–92.

20 GHO | By category | Global Information System on Alcohol and Health. WHO. http://apps.who.int/gho/data/node.main.GISAH?lang=en (accessed Nov 8, 2019).

21 OECD. Consumption Tax Trends 2018: VAT/GST and Excise Rates, Trends and Policy Issues. OECD, 2018 DOI:10.1787/ctt-2018-en.

22 Blecher E, Liber AC, Drope JM, Nguyen B, Stoklosa M. Global Trends in the Affordability of Sugar-Sweetened Beverages, 1990–2016. *Prev Chronic Dis* 2017; **14**: 160406.

23 Singh GM, Micha R, Khatibzadeh S, *et al.* Global, Regional, and National Consumption of Sugar-Sweetened Beverages, Fruit Juices, and Milk: A Systematic Assessment of Beverage Intake in 187 Countries. *PLOS ONE* 2015; **10**: e0124845–e0124845.

24 Ng M, Fleming T, Robinson M, *et al.* Global, regional, and national prevalence of overweight and obesity in children and adults during 1980–2013: a systematic analysis for the Global Burden of Disease Study 2013. *The Lancet* 2014; **384**: 766–81.

25 A century of trends in adult human height. *eLife*; **5**. DOI:10.7554/eLife.13410.

26 Alexandratos N, Bruinsma J. WORLD AGRICULTURE TOWARDS 2030/2050. 2012.

27 Goodchild M, Perucic A-M, Nargis N. Modelling the impact of raising tobacco taxes on public health and finance. *Bull World Health Organ* 2016; **94**: 250–7.

28 Basu S, Vellakkal S, Agrawal S, Stuckler D, Popkin B, Ebrahim S. Averting Obesity and Type 2 Diabetes in India through Sugar-Sweetened Beverage Taxation: An Economic-Epidemiologic Modeling Study. *PLoS Med* 2014; **11**: e1001582.

29 Shang C, Wang X, Slater S, Chriqui J, Lin Y, Chaloupka FJ. The pass-through of alcohol taxes to prices in OECD countries. 2018.

Supplementary Material

30 Cawley J, Willage B, Frisvold D. Pass-Through of a Tax on Sugar-Sweetened Beverages at the Philadelphia International Airport. *JAMA* 2018; **319**: 305.

31 Linegar DJ, van Walbeek C. The effect of excise tax increases on cigarette prices in South Africa. *Tob Control* 2018; **27**: 65–71.

32 Kostova D, Andes L, Erguder T, *et al.* Cigarette prices and smoking prevalence after a tobacco tax increase--Turkey, 2008 and 2012. *MMWR Morb Mortal Wkly Rep* 2014; **63**: 457–61.

33 Colchero MA, Salgado JC, Unar-Munguía M, Molina M, Ng S, Rivera-Dommarco JA. Changes in Prices After an Excise Tax to Sweetened Sugar Beverages Was Implemented in Mexico: Evidence from Urban Areas. *PLOS ONE* 2015; **10**: e0144408.

34 United Nations. Step by Step Guid to the Estimation Child Mortality. 2009.

35 Gardner JW, Stewart WH. Constructing Abridged Life Tables. 1966.

36 World Health Organization. Tobacco Free Initiative: Global Adult Tobacco Survey (GATS). 2018. http://www.who.int/tobacco/surveillance/survey/gats/en/.

37 Pearson AL, Cleghorn CL, van der Deen FS, *et al.* Tobacco retail outlet restrictions: health and cost impacts from multistate life-table modelling in a national population. *Tob Control* 2017; **26**: 579–85.

38 Blakely T, Cobiac LJ, Cleghorn CL, *et al.* Health, Health Inequality, and Cost Impacts of Annual Increases in Tobacco Tax: Multistate Life Table Modeling in New Zealand. *PLOS Med* 2015; **12**: e1001856.

39 Stacey N, Summan A, Tugendhaft A, Laxminarayan R, Hofman K. Simulating the impact of excise taxation for disease prevention in low-income and middle-income countries: an application to South Africa. *BMJ Glob Health* 2018; **3**: e000568.

40 Ozasa K, Katanoda K, Tamakoshi A, *et al.* Reduced Life Expectancy due to Smoking in Large-Scale Cohort Studies in Japan. *J Epidemiol* 2008; **18**: 111–8.

41 Gajalakshmi V, Peto R, Kanaka TS, Jha P. Smoking and mortality from tuberculosis and other diseases in India: retrospective study of 43[punctuation space]000 adult male deaths and 35[punctuation space]000 controls. *The Lancet* 2003; **362**: 507–15.

42 Zheng W, McLerran DF, Rolland BA, *et al.* Burden of Total and Cause-Specific Mortality Related to Tobacco Smoking among Adults Aged ???45 Years in Asia: A Pooled Analysis of 21 Cohorts. *PLoS Med* 2014; **11**. DOI:10.1371/journal.pmed.1001631.

43 Thun MJ, Carter BD, Feskanich D, *et al.* 50-Year Trends in Smoking-Related Mortality in the United States. *N Engl J Med* 2013; **368**: 351–64.

44 Doll R. Mortality in relation to smoking: 50 years' observations on male British doctors. *BMJ* 2004; **328**: 1519–0.

45 Gellert C, Schöttker B, Brenner H. Smoking and All-Cause Mortality in Older People. *Arch Intern Med* 2012; **172**. DOI:10.1001/archinternmed.2012.1397.

46 Kong L. A systematic review of cohort studies on the association of smoking with all-cause and lung cancer mortality in China. 2014. DOI:10.5353/th_b5320381.

47 Shavelle RM, Paculdo DR, Strauss DJ, Kush SJ. Smoking habit and mortality: a meta-analysis. *J Insur Med N Y N* 2008; **40**: 170–8.

48 Gu D, Kelly TN, Wu X, *et al.* Mortality Attributable to Smoking in China. *N Engl J Med* 2009; **360**: 150–9.

49 Lam TH. Mortality Attributable to Cigarette Smoking in China. *JAMA J Am Med Assoc* 1997; **278**: 1505.

50 Choi SH, Stommel M. Impact of Age at Smoking Initiation on Smoking-Related Morbidity and All-Cause Mortality. *Am J Prev Med* 2017; **53**: 33–41.

51 Dam RM v., Li T, Spiegelman D, Franco OH, Hu FB. Combined impact of lifestyle factors on mortality: prospective cohort study in US women. *BMJ* 2008; **337**: a1440–a1440.

52 Kenfield SA. Smoking and Smoking Cessation in Relation to Mortality in Women. *JAMA* 2008; **299**: 2037.

53 Jacobs DR. Cigarette Smoking and Mortality Risk. *Arch Intern Med* 1999; **159**: 733.

54 Brennan A, Meier P, Purshouse R, *et al.* The Sheffield Alcohol Policy Model - A Mathematical Description. *Health Econ* 2015; **24**: 1368–88.

55 Stockwell T, Zhao J, Panwar S, Roemer A, Naimi T, Chikritzhs T. Do 'Moderate' Drinkers Have Reduced Mortality Risk? A Systematic Review and Meta-Analysis of Alcohol Consumption and All-Cause Mortality. *J Stud Alcohol Drugs* 2016; **77**: 185–98.

56 Holmes J, Meier PS, Booth A, Guo Y, Brennan A. The temporal relationship between per capita alcohol consumption and harm: A systematic review of time lag specifications in aggregate time series analyses. *Drug Alcohol Depend* 2012; **123**: 7–14.

57 Singh GM, Micha R, Khatibzadeh S, *et al.* Global, Regional, and National Consumption of Sugar-Sweetened Beverages, Fruit Juices, and Milk: A Systematic Assessment of Beverage Intake in 187 Countries. *PLOS ONE* 2015; **10**: e0124845.

58 Finkelstein EA, Zhen C, Bilger M, Nonnemaker J, Farooqui AM, Todd JE. Implications of a sugar-sweetened beverage (SSB) tax when substitutions to non-beverage items are considered. *J Health Econ* 2013; **32**: 219–39.

59 Sánchez-Romero LM, Penko J, Coxson PG, *et al.* Projected Impact of Mexico's Sugar-Sweetened Beverage Tax Policy on Diabetes and Cardiovascular Disease: A Modeling Study. *PLOS Med* 2016; **13**: e1002158.

60 Wang YC, Coxson P, Shen Y-M, Goldman L, Bibbins-Domingo K. A Penny-Per-Ounce Tax On Sugar-Sweetened Beverages Would Cut Health And Cost Burdens Of Diabetes. *Health Aff (Millwood)* 2012; **31**: 199–207.

61 Hall KD, Sacks G, Chandramohan D, *et al.* Quantification of the effect of energy imbalance on bodyweight. *The Lancet* 2011; **378**: 826–37.

62 Ng M, Liu P, Thomson B, Murray CJL. A novel method for estimating distributions of body mass index. *Popul Health Metr* 2016; **14**: 6.

63 Veerman JL, Sacks G, Antonopoulos N, Martin J. The Impact of a Tax on Sugar-Sweetened Beverages on Health and Health Care Costs: A Modelling Study. *PLOS ONE* 2016; **11**: e0151460.

64 Aune D, Sen A, Prasad M, *et al.* BMI and all cause mortality: systematic review and non-linear dose-response meta-analysis of 230 cohort studies with 3.74 million deaths among 30.3 million participants. *BMJ* 2016; **9**: i2156–i2156.

65 World Bank. World Bank Country and Lending Groups. 2018. https://datahelpdesk.worldbank.org/knowledgebase/articles/906519.

66 WHO. Global status report on alcohol and health 2014. Geneva, Switzerland, 2014 DOI:/entity/substance_abuse/publications/global_alcohol_report/en/index.html.

67 Casetta B, Videla AJ, Bardach A, *et al.* Association Between Cigarette Smoking Prevalence and Income Level: A Systematic Review and Meta-Analysis. *Nicotine Tob Res* 2016; : ntw266.

68 Masood M, Reidpath DD. Effect of national wealth on BMI: An analysis of 206,266 individuals in 70 low-, middle- and high-income countries. *PloS One* 2017; **12**: e0178928.

69 Cobiac LJ, Tam K, Veerman L, Blakely T. Taxes and Subsidies for Improving Diet and Population Health in Australia: A Cost-Effectiveness Modelling Study. *PLOS Med* 2017; **14**: e1002232.

70 Tauchmann H, Lenz S, Requate T, Schmidt CM. Tobacco and alcohol: complements or substitutes? *Empir Econ* 2013; **45**: 539–66.
